# Towards Multiplex Molecular Diagnosis—A Review of Microfluidic Genomics Technologies

**DOI:** 10.3390/mi8090266

**Published:** 2017-08-30

**Authors:** Ismail Hussain Kamal Basha, Eric Tatt Wei Ho, Caffiyar Mohamed Yousuff, Nor Hisham Bin Hamid

**Affiliations:** Department of Electrical and Electronic Engineering, Universiti Teknologi PETRONAS, 32610 Seri Iskandar, Perak Darul Ridzuan, Malaysia; ismailhussain22@gmail.com (I.H.K.B.); cmd.yousuf@gmail.com (C.M.Y.)

**Keywords:** genomic diagnosis, microfluidics, multiplexed, point-of-care, lab on chip

## Abstract

Highly sensitive and specific pathogen diagnosis is essential for correct and timely treatment of infectious diseases, especially virulent strains, in people. Point-of-care pathogen diagnosis can be a tremendous help in managing disease outbreaks as well as in routine healthcare settings. Infectious pathogens can be identified with high specificity using molecular methods. A plethora of microfluidic innovations in recent years have now made it increasingly feasible to develop portable, robust, accurate, and sensitive genomic diagnostic devices for deployment at the point of care. However, improving processing time, multiplexed detection, sensitivity and limit of detection, specificity, and ease of deployment in resource-limited settings are ongoing challenges. This review outlines recent techniques in microfluidic genomic diagnosis and devices with a focus on integrating them into a lab on a chip that will lead towards the development of multiplexed point-of-care devices of high sensitivity and specificity.

## 1. Introduction

Infectious diseases are a major threat to human health as the leading cause of death worldwide [1]. Infectious diseases in humans are caused by out-of-control invasion of microorganisms like bacteria, viruses, fungi, protozoa, worms, and prions, collectively termed pathogens. Bacteria and viruses are responsible for 70% of human infectious diseases and 88% of epidemic outbreaks [2]. In 2010, contagious diseases such as measles, cholera, and meningococcal disease claimed 15 million lives [3] and are projected to cause 13–15 million deaths annually until 2030 [4]. The recent unanticipated Zika [5] and Ebola [6] epidemics highlighted a new risk of global contagion since infectious pathogens may now spread rapidly across large geographical distances because of enhanced human global mobility. There is deep concern over the lack of readiness of healthcare systems worldwide to contain and treat infectious outbreaks were they to occur.

To curb uncontrolled spread of infections, rapid identification, quarantine, and treatment of infected individuals is essential. Treatment towards cure can begin only after accurate identification of the infectious pathogen. For virulent agents, early identification is critical to maximize the infected individual’s chances of recovery. In an epidemic situation, any delay in response to an outbreak will exponentially elevate the risk of infection to the population through exposure and geographical spread. The number of people who need to be accurately screened, quarantined, and treated to contain the infectious outbreak grows quickly and, if local testing and quarantine resources fail to keep pace, the infection rapidly spreads out of control. 

Infectious agents can be identified from a biological sample (saliva, blood, urine, or stool) from the infected individual. Analysis of pathogens can be performed in specialized laboratories either by serology detection using enzyme-linked immunosorbent assays (ELISA) [7,8], morphology using electron microscopy [9,10], microbial culture [11], or molecular methods [12]. In ELISA, pathogenic antigens bind to a specific antibody linked to an enzyme and detected with a color change in the substance attached to the enzyme. Serological methods and morphological detection lack specificity and sensitivity because bacteria and virus share many features within families and serotypes, respectively [13]. Microbial culture is a non-routine, time-consuming, and research-based method in which the pathogen is detected by its induced characteristic changes in inoculated cells. Molecular methods require extraction of pathogen DNA or RNA, which is subsequently amplified and identified by sensitive and specific target probes conjugated with labels. In the lab, molecular methods are time-consuming and require specialized equipment and protocols.

In time-critical epidemic situations, lab-based diagnosis cannot meet the demands for timely identification of pathogens. The processing capacity of labs are limited because highly trained personnel must supervise the analysis and the biological sample must be transported from the patient at the point of care (POC) to the lab and results are usually obtained only after 24 h or longer [14]. In resource-limited settings, none of this infrastructure exists [15,16] and frequently the need for timely action against infectious outbreaks is more severe because of limited intervention and medication options. Diagnosis of the offending pathogen cannot be based only on observation of symptoms by medical professionals. Infected individuals may have variable delay in manifesting symptoms and most infectious bacteria and viruses cause similar symptoms like fever, weight loss, and respiratory problems. Collectively, these issues point to a crucial need for point-of-care tests to identify infectious agents rapidly and accurately. The World Health Organization (WHO) recommends the following criteria for such devices: (i) affordable; (ii) sensitive; (iii) specific; (iv) user-friendly; (v) rapid and robust; (vi) equipment-free; and (vii) deliverable to end-users [17,18].

POC tests are available commercially as diagnostic strips that utilize antigen and antibody detection principles, i.e., NS1 Bioeasy™, Anigen Rapid Rabies Ag Test kit (BioNote Inc., Gyeonggi-do, Republic of Korea) [19,20]. Although widely used, these test strips have a major limitation in that only one type of pathogen is tested per strip. The pathogen of interest must be identified prior to testing in order to choose the correct POC test. This precludes rapid and cost-effective identification of the offending pathogen in any new outbreak. For the same reason, the strips are also not feasible for routine healthcare practice as a low-cost and rapid diagnostic tool to identify infectious pathogens. For some diseases, diagnostic strips do not provide sufficient specificity (i.e., serotype) to inform treatment and can even be detrimental to the infected individual. In some diseases, antibodies against one serotype of virus may actually enhance the entry of a second viral serotype. This type of antibody-dependent enhancement has been observed between serotypes of the dengue virus [21] and also between dengue and Zika virus [22]. 

There is a clear need for next-generation POC tests capable of multiplex detections in a single test with genomic-level specificity. We believe this is the crucial feature that will motivate widespread use of POC tests for rapid disease identification in epidemics as well as routine medical diagnostics of infectious diseases. Microfluidics technology has promising techniques to automate multiplex genome detection on a chip with the attendant advantages of reduced biological sample and reagent volume, speedup and simplification of diagnostic processing, and reduced diagnostic errors from sample contamination [23]. Test devices are portable and disposable and have the potential to meet the WHO criteria for POC tests.

This review focuses on the recent advances in genomic diagnosis techniques implemented in microfluidic chips. Through examination of deoxyribonucleic acid (DNA) or ribonucleic acid (RNA) extracts from the pathogens in the biological sample, infectious pathogens can be identified with high specificity. This process involves several key steps [16], namely: 1. lysis (disruption of the cell or viral wall to expose genetic material); 2. nucleic acid extraction (separation and purification of DNA/RNA from other sub-cellular components); 3. amplification of specific DNA/RNA; and 4. matching the genetic material to reference pathogen sequences. We review various microfluidic methods that have demonstrated each or a combination of these four component steps. Finally, we discuss possible future directions towards a fully automated, multiplexed lab on a chip system for pathogen diagnosis with high sensitivity and specificity. 

## 2. Lysis Techniques

Lysis is the first and most crucial step in disrupting the cell membrane or cell wall to extract genomic material for pathogen identification. Lysis can be performed through chemical [24], mechanical [25], electrical [26], or thermal [27] means. The choice of method depends on the structure of the pathogen envelope. The nucleic acids of mammalian cells are enclosed within a cytoplasmic membrane. In viruses, nucleic acids are enclosed within a protein shell called a nucleocapsid and in some of the virus families like Herpesvirus or Flavivirus, the nucleocapsid is further encased within a glycoprotein envelope. Bacterial nucleic acids are enclosed within a cytoplasmic membrane that resides inside the cell wall. Some species of bacteria have an additional outer polysaccharide capsule. Gram-negative bacteria have a cell wall comprising a thin layer of peptidoglycan between the inner and outer lipid membranes. Gram-positive bacteria have a cell wall composed of a thick layer of peptidoglycan and lipoteichoic acid surrounding a single lipid membrane. It is relatively easier to lyse mammalian cells compared to bacterial cells due to the additional cell wall. 

Biological samples have diverse chemical environments and content. The pH, ionic strength, conductivity, and presence of molecular inhibitors are possible obstructions to lysis. Moreover, the lysis process may damage the integrity of nucleic acids that are released from the cell or virus. Care must be taken to tune the lysis process to avoid excessive degradation of genomic material, which will impede accurate identification. 

### 2.1. Chemical Lysis

Chemical lysis relies on solutions or enzymes to dissolve the membrane or wall of the pathogenic cell. The choice of buffers and enzymes are dependent on the type of cell and sample medium. Detergents like sodium dodecyl sulfate (SDS), sodium deoxycholate, nonyl phenoxypolyethoxyl ethanol (NP-40), or Triton-X are common solutions that dissolve phospholipids [28], which are the building blocks of mammalian cell membranes [29]. For example, a typical buffer mixture could contain Tris-HCL, Triton X-100, and Tween-20 mixed with a protease [30]. Maslov et al. [31] designed an array of microwells that entrapped up to three HeLa cells for lysis using a buffer containing Triton-X and EDTA. The lysis process was completed within 12 s. Apart from detergents, chaotropic agents are also used to lyse mammalian membranes by solubilizing the hydrophobic region in the phospholipids, thus causing disruption of the membrane. 

**Figure 1:**
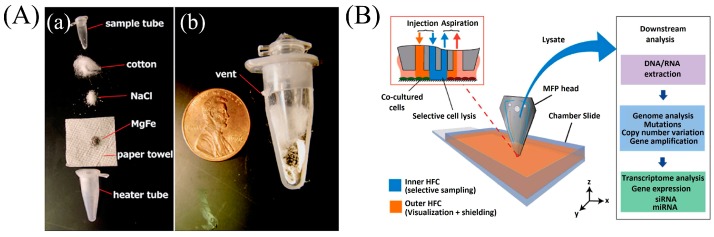
Chemical lysis. (**A**) Chemical heater: (**a**) exploded view of heater tube, paper towel with MgFe, NaCl, and cotton. Water was added to trigger heating. (**b**) Assembled sectional view of heater. Adapted from [35] with permission of The Royal Society of Chemistry (**B**) Selective lysis of cells: Illustration of a microfluidic probe head skim through cells, the interaction of biochemicals with the cells (inset). Reprinted from [40] with permission of Nature Publishing Group, Copyright 2016.

Bacterial and yeast cell walls are composed of other biological polymers, like cellulose, pectic polysaccharides, and phenolic compounds [32], which can be lysed by lysozyme enzymes mixed with ethylenediamine tetraacetate (EDTA). Lysozyme digests the peptidoglycan by breaking the glycosidic bond, while EDTA protects against DNA degradation by inhibiting the activity of cellular enzymes [33] by binding with metallic cations. Cichova et al. [34] developed a continuous flow biochip for online bacterial cell lysis and DNA extraction using 1% Triton X-100 and 5% Chelex. Chelex was added to the lysis buffer to protect the DNA from degradation by the DNases. Achromopeptidase (ACP) is used with gram negative bacteria instead of lysozyme to lyse the thicker outer membrane along with cell wall. Cells will sometimes cluster into tissue and the protease enzymes—trypsin or collagenase—are used to disaggregate and dislodge individual cells. Buser et al. [35] used dried ACP to lyse gram-positive bacteria (*Staphylococcus aureus*) and RNA virus (respiratory syncytial virus) in a minute with the aid of a disposable chemical heater, as shown in Figure 1A, to control deactivation of the ACP enzyme. 

Lysis buffers designed to protect the integrity of DNA and RNA from degradation are commercially available like PureZOL™ RNA Isolation Reagent [36]. For DNA extraction, the buffers may contain sodium hydroxide and SDS. Hydroxide ions slice the fatty acid–glycerol ester bonds of phospholipids in the cellular membrane, thereby releasing intracellular material. In one example, hydroxide ions were electrochemically generated by electrolysis of a saline solution [37,38]. This method is not suitable for RNA extraction because the RNA backbone is hydrolysed in an alkaline environment [37]. Instead, buffers for RNA extraction may contain phenol, guanidine isothiocyanate, and RNase inhibitors. For compatibility with some downstream methods, like isotachophoresis for DNA/RNA separation, special lysis buffers may be required to maintain a specific electrolyte content, pH, ionic strength, and solution conductivity. 

There are few recipes for selective lysis to extract genomic material only from a particular group of pathogens in a diverse biological sample. Zelenin et al. [39] lysed all blood cells using 1% saponin but left the bacteria cells intact for analysis as the bacterial cells are unaffected by Triton X-100 and saponin. Kashyap et al. [40] developed a scanning microfluidic probe to selectively lyse cells in adherent cultures. The probe injects sodium hydroxide over cells of interest using advanced hydrodynamic manipulation in order to lyse and subsequently extract the DNA and RNA that is released (Figure 1B).

Often, microfluidic chemical lysis needs an additional mixing step to diffuse the lysing chemicals into the cells. Fluidic mixing does not occur naturally at microfluidic scales when laminar flow is used [25] and must be forcefully achieved with microfluidic mixers [39,41]. The lysing chemicals must also be removed prior to subsequent genomic analysis to avoid inhibiting the subsequent extraction and amplification processes [37]. For example, 0.01% of the SDS lytic reagent inhibits the polymerase chain reaction (PCR) process during sample amplification. Additional steps are required to remove or dilute the lytic chemicals like washing and elution steps in extraction process [42]. Dilution of chemicals is a poor choice because it reduces the detection sensitivity of genome identification.

### 2.2. Mechanical Lysis

Mechanical lysis techniques perforate the cell membrane with a sharp cutting edge or the application of high pressure. Mechanical lysis can be achieved through four types of cutting motions: shearing, shocking, grinding, or beating. 

**Figure 2:**
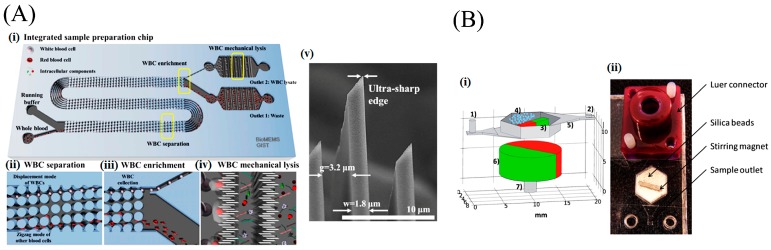
Mechanical lysis. (**A**) Integrated WBC enrichment and lysis: (**i**) a schematic illustration of WBC separation and mechanical lysis; (**ii**) lateral displacement of WBCs; (**iii**) self-enrichment of WBCs by controlling the width ratio between two outlets; (**iv**) WBCs are simultaneously ruptured by mechanical nanoblade arrays; (**v**) Magnified image of ultra-sharp edge. Reprinted from [45] with permission of Nature Publishing Group, Copyright 2015. (**B**) Bead beating mechanical lysis device: (**i**) Schematic of components: (**1**) inlet, (**2**) outlet, (**3**) stirring magnet, (**4**) zirconia/silica beads, (**5**) bead weir, (**6**) rotating magnet, and (**7**) electric motor coupling; (**ii**) Digital Image of lysis device. Reproduced from [25] with permission of The Royal Society of Chemistry.

Lysis using a shearing mechanism is demonstrated using nano-bars and nano-spikes. The cellular membrane is disrupted when they pass through the sharp opening. Yun et al. [43] fabricated an array of nano-spokes, with 2 μm spoke spacing and tip diameter of 10 nm, by wet-etching crystalline silicon. They lysed EL4 T-lymphoma cells for protein extraction in under 2 min, which is comparable to that of conventional chemical lysis. Enrichment of cells is also useful in lysing [44]. Later, Choi et al. [45] of the same group used deterministic lateral displacement (DLD)-based sorting and lysed white blood cells (WBCs), separated from the whole blood without any sample dilution (Figure 2A). DLD-based self-enrichment improved the efficiency to 75%. Kim et al. [46] constructed inexpensive ZnO nanowires compared to nano-spokes to lyse HaCaT, HeLa, and Jurkat cells. The cells are anchored and lysed by rupturing the membrane due to sharp Zinc oxide (ZnO) tips and also by the fluid-induced shear force. However, the Jurkat cells with a diameter of 10 μm escape from the nanowires with a gap of 15 μm, resulting in inefficient lysis. The nano-spoke array is attractive for lysis of larger mammalian cells with a diameter of 13 μm and above. However, the bacteria or nanometer-sized viruses can easily escape through the micrometer spoke spacing. 

Porous polymeric monoliths (PPMs) also utilize a shearing mechanism in the disruption of the cell wall and membrane, as the cell passes through microscopic pores. PPMs were used by Mahalanabis et al. [47] to lyse bacterial cells and Burke et al. [48] to lyse white blood cells using PPM formed in a polydimethylsiloxane (PDMS) device. Aly et al. [49] used antimicrobial polymers along with PPM to lyse *E. coli* and B. subtilis. The antimicrobial polymers have the ability to inhibit microbial organisms by killing them. The same group later improved the efficiency of lysis (89%) by regulating the hydrophobic–hydrophilic property of the PPM [50]. They also suggest that use of PPM did not inhibit the PCR amplification process. 

Bead-based cell lysis uses frictional forces from collision with the beads to disrupt the membrane. Geissler et al. [51] experimented bead-based lysis for *Bacillus atrophaeus* subsp. The operation of the chip is based on beating and agitation. The chip is fabricated using machined slides of polymethylmethacrylate (PMMA) and firms a metal disk along with solid micro beads in a lysis chamber. Magnetic forces are used to actuate the disk to induce collision and friction forces on the cell membrane by colliding micro beads with the cells for lysis. Four lysis processes are completed in parallel with an efficiency of 85% in 10 min. Similarly, Berasaluce et al. [25] used bead beating features and a magnetic stirrer for cell wall disruption of *Staphylococcus epidermidis* bacteria, as shown in Figure 2B. They experimented with the lysis efficiency based on four parameters: bead size, bead quality, percentage of Tween 20 used, and flow rate. The usage of small beads and 50% quantity increased the amount of DNA released. Usage of Tween 20 increases DNA recovery by denaturing the cell wall. Also, at a low flow rate, the lysis efficiency increases. With 100 μm bead size, 45% bead quantity, and 0.05% Tween 20, the efficiency was 56% at a flow rate of 30 μL·min^−1^. 

Recently, Cheng et al. [52] demonstrated cell disruption and sample transport using an on-chip micropump. The cell disruption is carried out using peristaltic deformation of PDMS membrane. The deformed membrane closes the annular channel and disrupts the cells using collision and frictional forces. A 50-μL cell sample is lysed in 36 s with 80.6% and 90.5% cell disruption rates for the HEK293 cell sample and human natural killer cell. However, multiple time disruptions (30 cycles) are needed for better disruption rates.

**Figure 3:**
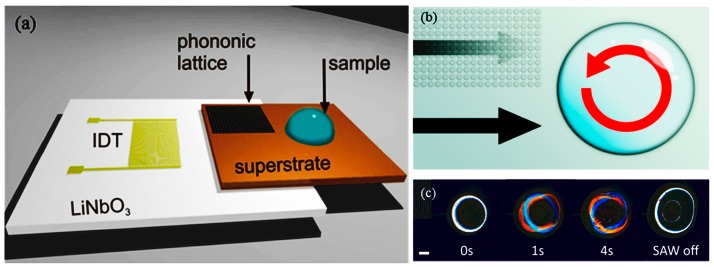
Surface acoustic wave (SAW) stage used to lyse cells: (**a**) interdigitated transducer (IDT) used to generate the SAW; (**b**) the phononic lattice absorbs SAWs in a frequency-dependent manner. This acts to create a rotational movement within the droplet and results in shear flows that contribute to the disruption of the cell membrane. (**c**) The sequence of images shows a droplet of cells undergoing SAW-induced mechanical lysis. Reprinted with permission from [53]. Copyright (2015) American Chemical Society.

In acoustic lysis, (cavitation) bubbles are generated using ultrasonic excitation, and shock waves from a collapsed bubble may lyse the cell wall and membrane. In acoustic streaming, high-intensity acoustic fields generate a time-independent flow (Vortical flow) to create a viscous drag on the cell walls/membrane and shear them. Reyhani et al. [53] demonstrated surface acoustic waves (SAW) lysis capable of lysing cells from a human cancer cell line. A 20-μL droplet holding the cell suspension is placed as shown in Figure 3 and shear-induced flow is generated in droplets using SAW, resulting in lysis. The efficiency of SAW lysis is 12.9% ± 0.7%. Absorption of acoustic energy generates heat at durations exceeding 50 s, which could damage the intracellular molecules. Recently, Wang et al. [54] used a surface acoustic streaming wave for cell lysis by accelerating high-speed collision of cells towards SU8 micro-pillars, on a LiNbO3 substrate. The heating problem is elevated by maintaining the temperature below 40 °C using an aluminum heat sink. Ninety-five percent of cell lysis is achieved within 20 s, with proof of the nucleic acid integrity. Similarly, Taller et al. [55] used (SAWs) to lyse exosomes from raw cell media with an efficiency of 38%. 

Though mechanical lysis techniques are reagentless, their application is limited by cell size. The gap between nano-spokes and PPM pore diameter limits the usage of this technique in lysis of viruses. Moreover, construction of sharp scale bars requires a complex fabrication process. Alternatively, bead-based lysis poses a threat of integrity of nucleic acids, as the collision of beads will damage the nucleic acids [56].

### 2.3. Electrical Lysis

Electrical lysis utilizes a high electric field to induce dielectric breakdown of the cell. The principle of electrical lysis is when an external electrical field is applied towards the cell, a potential difference, i.e., induced transmembrane potential, is generated across the cell membrane. When this induced transmembrane potential exceeds a threshold value, the phospholipids on the membrane reallocate due to permeability and the electric dipole moment (phosphate group—electropositive and hydrogen chains—electronegative), causing the membrane to disrupt.

**Figure 4:**
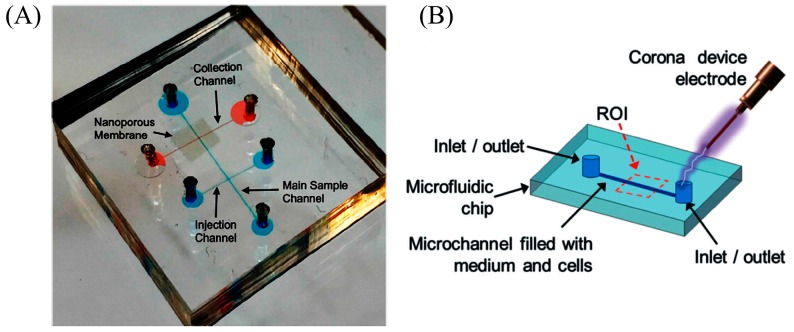
Electrical lysis: (**A**) electrical lysis using nanoporous membrane; Reproduced with permission from [60]. (**B**) Schematic of the experimental setup using corona discharge. Reproduced from [61] with permission of The Royal Society of Chemistry.

The problem associated with macro-scale electrical lysis is the need for high voltage to lyse the cell. Micro-fabrication techniques made it possible to fabricate microelectrodes and reduce the spacing between electrodes, therefore electrical fields up to MV/m can be easily generated using portable battery supplies, and these electric fields are sufficient to disrupt the cell membrane. In addition, the electrical field can be intensified by manipulating the channel geometry in a certain section of the channel to realize electrical lysis [57,58]. Yet attractively, 3D microelectrodes are also used to intensify the electric fields. Gabardo et al. [59] used 3D structurally optimized microelectrodes decorated with nanoscale features to lyse *E. coli* cells with 4 V with an efficiency of 95%. Recently, Islam et al. [60] used commercially available nanoporous membranes crammed between two microfluidic channels (Figure 4A) to generate thousands of parallel nanopores for trapping and to generate a highly localized electric field for lysis. A lysis efficiency of 90% is obtained for *Escherichia coli* (*E. coli*) with 0.4 μm pore size. However, the pore size determines the lysis efficiency: *E. coli* with 0.5 μm will easily pass through 0.5 μm pores and a pore size of less than 0.2 μm will hinder the continuity of electricity and flow.

The major problem in electrical lysis is Joule heating, which denatures the nucleic acids. The Joule heating can be reduced by using pulsating direct current (DC) field and alternating current (AC) field in micro-fabricated electrodes. DC voltages generate unwanted electrochemical reactions at the electrode–electrolyte boundary. For example, gas and bubbles generated during the lysis process can block the channel. In addition, the targeted biological molecules will be degraded by the electrochemical products of redox reactions at the electrode–electrolyte interface [62]. Moreover, redox reactions can also damage the electrodes. The pH and ionic composition of the lysate drastically changes. To address this problem Talebpour et al. [62] developed surface-enhanced blocking electrodes (SEBE) that are capable of evading unwanted redox reactions. The principle is to use a thin dielectric coating at the electrode and electrolyte interface, thus establishing blocking electrodes. SEBE were also used to suppress the rapid harassing electric fields in the cell suspension medium. Similarly, indium tin oxide (ITO) electrodes generate unwanted faradaic reaction effects in cells [63] and ITO can be replaced with graphene. 

In another form, corona discharge can also be used to generate electric fields in the kV/cm range without generating heating and bubble formation. The theories behind corona discharge in liquids are diverse. Escobedo et al. [61] used a handheld corona device, as shown in Figure 4B (electric discharge of 10 to 30 kV: 300 ms), to lyse baby hamster kidney cells (BHK), enhanced green fluorescent protein human-CP cells (eGFP HCP) with an embedded microelectrode, and non-adherent K562 leukemia cells without microelectrodes.

Electric fields for lysis can also be induced optically, i.e., optoelectronic tweezers (OET). Upon illumination with a laser beam, local electric field gradients are generated around the photoconductive material and such fields have been sufficient for single cell lysis. Witte et al. [64] used an amorphous silicon (aSi) photoconductor as a virtual electrode. A single red blood cell (RBC) is lysed in 1 min using a 2.5 μm diameter beam. A large cluster of RBCs are also lysed within 80 s. Huang et al. [56] integrated optically induced cell lysis (OICL) and optically induced dielectrophoresis (ODEP) to lyse a human embryonic kidney cell and extract the nucleus from it. The OICL has a lysis efficiency of 78.04 ± 5.70%. In OICL, programmed light patterns are digitally projected to function as virtual electrodes. This technique has the ability to select single cells and lyse them.

In addition, electrical lysis was found to be compatible for further downstream analysis. Geng et al. [65] used pulse electric field to lyse both eukaryotic cells (CHO cells) and bacterial cells (*Salmonella typhimurium* (*S. typhimurium*)). Magnetic beads are used to extract DNA from the cell lysate. The chip contains 100 μm wide gold electrodes separated by a distance of 3 mm. The extraction efficiency of DNA was up to ~36% for CHO cells and up to ~45% for *S. typhimurium* and is comparable to the chemical lysis technique. Similarly, Ma et al. [66] used microscale silica beads to trap the bacteria and electrical lysis. It has also been shown that mRNA extraction efficiency was 10–20 times greater than available mechanical bead beating methods.

Although the use of pulsating electric fields reduces the joule heating, the design and construction of low cost nanosecond pulsating circuits are challenging as the amount of electric field needed to lyse depends on the cell size. For the same device configuration, the voltage needed to lyse nanoscale viruses is higher than that for microscale bacteria. Moreover, the reusability of gold and ITO electrodes is disputed, thereby increasing the cost of POC devices.

### 2.4. Thermal Lysis Techniques

Thermal lysis relies on high temperature (90–100 °C) to denature proteins in the cell membrane [67]. In microfluidics, thermal lysis is performed either by ohmic heating or by placing the chip on the hot plate. A serpentine microfluidic thermal lysis device was developed by Packard et al. [27] for *E. coli* lysis in less than 60 s with 65 °C. They attached a simple resistive heater and controlled the temperature externally. They suggested that increasing the temperature intensity and duration will increase the percentage of lysis. However, elevated temperatures denature a majority of non-DNA biomolecules and RNA within the cell. DNA gets denatured at approximately 95 °C and RNA gets degraded at >65 °C. Selective capture and lysis is demonstrated by Tsougeni et al. [68]. They used anti-*Salmonella* antibodies precoated on a plasma nanotextured cell capture module for trapping cells (with an efficiency of 80–100%) and demonstrated on-chip thermal lysis down to 10 bacterial cells.

## 3. Nucleic Acid Extraction

After lysis, genomic material from within cells or viruses is released, together with membrane debris and various polysaccharides, metabolites, and ions. The lysate contains inhibitors to nucleic acid amplification [69], nuclease to degrade nucleic acids (by cleaving the phosphodiester bonds), and, for best results, DNA and RNA must be separated from the lysate prior to amplification and detection. The success of downstream processing and detection sensitivity hinges on the quantity and purity of nucleic acid extracted for amplification [70]. 

### 3.1. Solid Phase Extraction

Solid-phase extraction (SPE) systems utilize functionalized surfaces as vehicles to collect genomic material. The SPE uses silicon micropillars, silica beads, or functionalized magnetic beads to capture the nucleic acids. The beads are larger in size and easier to manipulate. Nucleic acid extraction proceeds through a three-step process: the target nucleic acids bind to functionalized beads, impurities are washed out while the DNA and RNA remain tethered to the beads, and nucleic acids are eluted from the beads. 

In silica bead SPE systems, the silica particles are arrayed in a porous polymer monolithic. The principle of silica-based SPE is formulated based on the binding properties of nucleic acids with the silica catalyzed by a chaotropic agent. A salt bridge is formed between nucleic acids and silica particles for binding. Subsequently, nucleic acids are washed with ethanol and higher concentration salts and eluted with lower concentration salts. Guanidine is widely used as a chaotrope for high-efficiency DNA extraction, even though it is toxic in nature and guanidine waste is an environmental contaminant. Also, it has been proven that guanidine act as an inhibitor in PCR amplification, so Chen et al. [71] proposed a sodium chloride (NaCl)-based extraction method to extract genomic DNA from biological samples. NaCl is a natural, cheap, and non-toxic green reagent to replace guanidine. Compared with guanidine, NaCl is able to extract 667.1–1181.1 ng/mL DNA from the whole blood of a rat in 30 min. Similarly, Hagan et al. [72] used a chitosan-based RNA binding phase for extraction of nucleic acids. Chitosan is used to avoid the PCR inhibitory effects caused by guanidine and isopropanol. 

The challenging procedure in microfluidic silica SPE is to organize and pack uniform beads inside the microchannel. In this context, Hwang et al. [73] developed a built-in flexible PDMS membrane to pack beads. The flexible membrane controls the surface-to-volume ratio (SVR) of packed beads with different bead amounts, which is used for the reliable operation and to enhance the cell capture efficiency. This device is used to extract nucleic acids from methicillin-resistant *Staphylococcus aureus* (MRSA) in nasal swabs. The overall extraction process took less than 20 min.

Magnetic beads are widely used because the beads can be collected or positioned through external magnetic fields, have good thermal resistance, and do not react with the various chemicals used during lysis [74,75]. Silica-coated paramagnetic beads, oligo (dT)-coated beads, and sequence-specific paramagnetic beads are reportedly used. Silica-coated paramagnetic beads are non-specific in nature and operate on the same principle as silica-based SPE, but are limited in downstream application because of the requirement of a chaotropic agent. 

The extraction efficiency of magnetic beads hinges on the specificity of nucleic acids binding to the surface of the beads. Oligo (dT) (deoxy-thymidine oligonucleotides) beads are specifically preferred to extract messenger RNA (mRNA), which contains useful information on the activities of specific populations. Polyadenosine (poly-A) tails are specifically present in mRNAs and Oligo (dT) bind only to a poly-A tail and not to general nucleic acids. Han et al. [76] proposed a microchip consisting of magnetic oligo-dT beads in a microchannel for extracting mRNA from the lysate of biological samples. The magnetic beads and mRNA are binded off the chip by using pipetting steps. The device adds in ferromagnetic wire arrays to generate a high gradient magnetic field to separate the lysate from the magnetic beads hemmed in by mRNA. Similarly, in sequence-specific beads, precise oligonucleotide sequences are coated on the beads surface for specific extraction. Sequence-specific beads are preferred for the extraction of microRNAs because of their short sequence [77]. 

**Figure 5:**
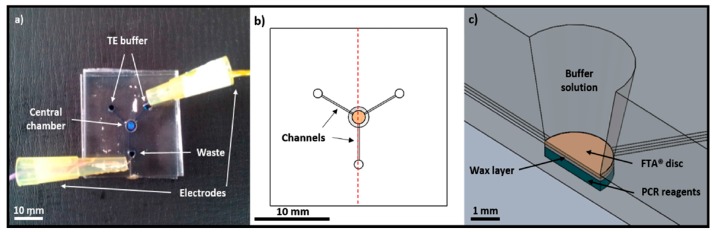
(**a**) Microfluidic device used to perform integrated DNA purification and amplification experiments; (**b**) schematic top view of the channels and central chamber; (**c**) schematic cross section view of FTA^®^ paper discs placed on top of a layer of wax-encapsulated PCR. Reproduced with permission from [80].

Flinders Technology Associates (FTA) filter papers are also used in the purification of nucleic acids without the need for an elution step. Filter papers have been used as a processing substrate to destroy non-genomic material and simultaneously hold DNA and RNA. The filter paper is a cellulose membrane with absorbed denaturing agents, chelating agents, and traps for radicals. Some reports claim that the FTA filtration membrane binds the primers, which inhibits the amplification reaction. FTA membranes also exhibit autofluorescence, which interferes with the fluorescence generated by genomic material during detection [78]. Several groups have developed workarounds. Liu et al. [79] and Wimbles et al. [80] also loaded amplification reagents onto FTA filters. Liu demonstrated extraction and isothermal amplification of viral nucleic acid, while Wimbles incorporated electro-osmotic flow (EOF) to transport washing buffers to the membrane (Figure 5).

However, the solid phase extraction needs a special coating of magnetic beads, monolithic porous structures, and packed beads for operation. Though SPE is well established, the need for pumping reagents for multiple wash steps and the usage of permanent magnets increases the chip area. Furthermore, the SPE binding capacity is limited to protein absorption. The use of isopropyl alcohol, guanidinium thiocyanate (GuCN), guanidinium chloride (GuHCl), or ethanol limits the operation of POC devices due to their inhibiting effects. In addition, magnetic beads are costly, which limits their usage in resource-limited settings. 

### 3.2. Isotachophoresis

Isotachophoresis (ITP) is an electrokinetic approach that uses differences in electrophoretic mobility between DNA, RNA, and other lysates to extract and concentrate genomic material. An external electric field is applied across a microfluidic channel to induce motion of the lysate solution, which is placed between a leading (LE) and trailing electrolyte (TE). The choice of leading and trailing electrolyte is a key design parameter. The electrophoretic mobility of the nucleic acids must lie between the mobility of both electrolytes, and other lysates should have lower mobility than TE. Electrophoretic mobility varies with the charge content and hydrodynamic size of the nucleic acid [69]. A comprehensive review of isotachophoresis and the properties of various lysates can be found in [81]. The values of LE and TE mobility can be obtained from simulation software like Peak Master, SIMUL, SPRESSO, Buffer Calculator, STEEP, and Ionise [82]. However, the design of microchip and separation length requires knowledge of inhibitors, which changes with the concentration of complex samples and their dilutions, thereby limiting the use of the same design in a variety of samples. 

**Figure 6:**
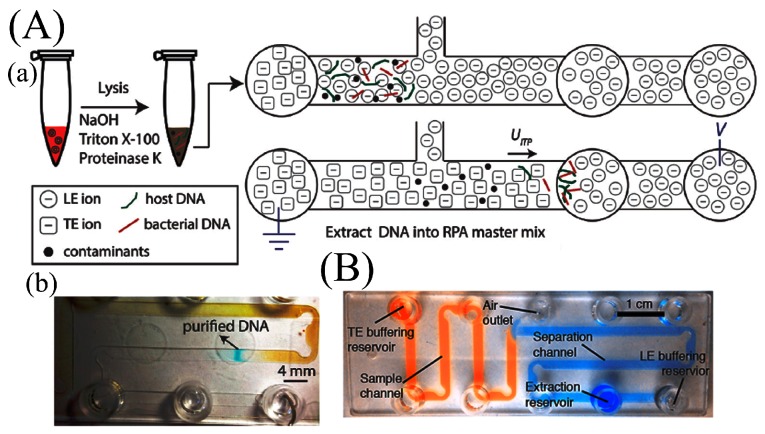
Microfluidic isotachophoresis device. (**A**) Schematic of ITP compatible lysis: (**a**) schematic of electric field configuration for ITP; (**b**) ITP extraction process in chip. Purification and separation of DNA from whole blood contaminants. Adapted from [70] with permission of The Royal Society of Chemistry. (**B**) Microfluidic device to improve extraction efficiency. Reprinted from [91]. Copyright (2014), with permission from Elsevier.

In 1975 Brocek et al. [83] demonstrated the first on-chip ITP separation of six carboxylic acids, and ITP extraction of nucleic acid extraction was first demonstrated by a group of cancer researchers in Moscow [84,85]. ITP has been integrated with a variety of lysis and amplification methods and applied to various types of pathogens. Nucleic acids have been extracted from whole blood [86], cell culture [87], and urine [88] lysates. Rogacs et al. [89] demonstrated 16S rRNA extraction from *Pseudomonas putida*-infected human blood cells followed by PCR. They achieved a 6-fold increase in sensitivity compared to solid phase extraction but only 0.2% extraction efficiency with 5 min processing time. Marshall et al. [90] applied thermal lysis with ITP on a printed circuit board (PCB) to extract genetic material from *Plasmodium falciparum* and achieved a detection limit of 500 parasites/μL. Marshall et al. [91] demonstrated improved recovery efficiency up to 81% of DNA from whole blood with a 20 min extraction time. The device shown in Figure 6B incorporates larger width (2 mm) and length (0.15 mm) to reduce the Joule heating effect in the purification and extraction of nucleic acids from 25-μL biological samples. The design also includes optimized turn geometries to reduce the sample dispersion. Moreover, buffering TE and LE are added to aid the extraction efficiency by maintaining pH. Eid et al. [70] verified the same design to amplify DNA from *L. monocytogenes* cells spiked into whole blood (Figure 6A). Shintaku et al. [92] demonstrated electrical lysis with ITP to separate RNA and DNA from single lymphocyte cells. The same group [93] subsequently showed on-chip simultaneous analysis of cytoplasmic RNA and genomic DNA (gDNA), also from single cells. The RNA and gDNA were separated into two different outlets utilizing sequence-specific electric fields within a 5-min extraction time. The simultaneous correlative analysis of RNA and DNA is useful in cancer-related research, diagnosis, and in genetic manipulations [94,95]. 

## 4. Amplification

Amplification of genomic material is required prior to detection because the concentration of nucleic acids extracted is usually insufficient for direct detection. Amplification-free detection techniques have been reported in the literature, like plasmonic sensing [96], antiresonant reflecting optical waveguide (ARROW) [96], and electrochemical detection [97], but these methods are infeasible for POC application due to the complex fabrication procedures, dependence on target labeling, and low detection sensitivity [96]. Nucleic acid amplification requires regulated temperature. Isothermal amplification operates at a single temperature, whereas non-isothermal amplification requires multiple temperature zones at different processing steps [98]. The sensitivity and specificity of amplification depend on the design of primers and binding of the primer to the template. 

### 4.1. Cyclo-Thermal Amplification

Polymerase chain reaction (PCR) was invented by Kary Mullis in the 1980s [99] and is the most popular technique for the amplification of specific DNA. The main components of PCR reagents are DNA polymerase, two sets of primers (oligonucleotides), and deoxynucleoside triphosphates (dNTPs). DNA polymerase is an enzyme that polymerizes new DNA strands to the template DNA. There are two sets of primers: reverse primers that hybridize to template ssDNA and forward primers that hybridize to the complementary ssDNA. Primers provide the required binding site for DNA polymerase. dNTPs are basic elementary units for the DNA polymerase to create a new DNA strand. With DNA as the starting template, PCR involves three steps of thermal cycling: denaturation of double-stranded DNA (dsDNA) into single-stranded DNA (ssDNA) at 95 °C, annealing of primers to ssDNA at ~55 °C, and enzymatic elongation of the annealed primers at 72 °C. These thermal cycles are repeated until there are sufficient copies of the nucleic acids for detection. Temperature can be cycled in a stationary chamber or the PCR solution could be continuously flowed (pump or convection) through reaction chambers held at different temperatures [100]. Therefore, theoretically more than 1 billion DNA molecules of interest can be generated after 30 cycles from a single double standard DNA target region. To amplify RNA, it is first converted into complementary DNA (cDNA), followed by reverse transcription PCR (RT-PCR). Fluorescent dyes may be mixed into the PCR solution to monitor copy progress in real time and provide useful information to determine the initial number of DNA copies (quantitative RT-PCR). In digital PCR (dPCR), the PCR solution is partitioned into several small volume reaction chambers to be used for absolute quantification of the target copy number.

#### 4.1.1. Stationary Chamber Polymerase Chain Reaction (PCR)

The first stationary chamber-based PCR chip was demonstrated by Northrup et al. in 1993 [101]. The rate-limiting step in stationary PCR is arguably the rate of heating and cooling to transition between thermal cycles. In a simulation study comparing a stationary chamber with continuous flow PCR (CF-PCR) devices, Papadopoulos et al. [100] found that stationary PCR required longer processing times (up to twice as long) compared to CF-PCR due to temperature transitions. Recent developments in stationary PCR focus on the use of special primers, novel heating methods, and the integration of multiple genome detection methods (multiplexing PCR) to reduce thermal cycling time. Houssin et al. [102] demonstrated ultra-fast real time PCR (30 cycles in 2 min) by circulating pre-heated liquids in a microfluidic chip without losing detection sensitivity. Farrar et al. [103] observed that the time required for temperature cycling is inversely proportional to the primer and polymerase concentration, but at the cost of decreased sensitivity and specificity due to greater probability of mispriming. They demonstrated a capillary-based fast PCR with reduced cycling time (0.4–2 s) by increasing the concentration 10- to 20-fold with a simple hot bath for temperature cycling. They succeeded in amplifying a 60-bp genomic target in 14.7 s with 35 cycles, attaining single-molecule sensitivity and 91.7–95.8% efficiency of amplification.

**Figure 7:**
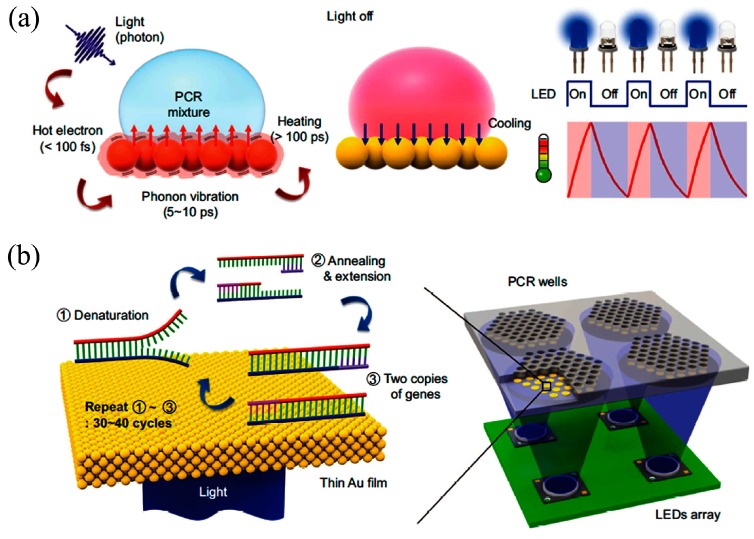
Ultrafast photonic PCR (**a**) Schematic of the plasmonic photothermal induced heating of the PCR mixture; (**b**) schematics of the ultrafast photonic PCR using a thin gold (Au) film cycling with LED arrays. Reprinted from [108] with permission of Nature Publishing Group, Copyright 2015.

Several researchers showed the potential for fast thermal control through optical heating methods. Roux et al. [104], Pak et al. [105], Lounsbury et al. [106], Liu et al. [107], and Hagan et al. [72] used infrared (IR)-mediated heating for RT-PCR amplification to reduce amplification time. In a representative example from Hagan, the analysis time for RNA-based influenza A virus decreased 5-fold (to 39 min). Son et al. [108] achieved thermal ramping from 55 °C (annealing) to 95 °C (denaturation) within 5 min by employing thin Au films that convert incident LED light into heat through plasmon-assisted optical absorption (Figure 7). Phaneuf et al. [109] presented thermal multiplexing for multiplexed PCR using a laser diode, a single radiative heat source that is selectively modulated for temperature control in an array of PCR reactions. The system is able to generate different annealing temperatures in a single microchip suitable for simultaneous amplification 500 bp and 600 bp amplicons. The amplification of λ-phage (annealing at 68 °C for 500 bp) and EBV template (annealing at 48 °C for 600 bp) DNA is performed in 110 min. 

Qiu et al. [12] reported a convective polymerase chain reaction (CPCR) for rapid molecular detection of H1N1 virus on a dipstick assay. A fully disposable chemically heated thermal processor is established to heat the capillary tube from the bottom at a fixed temperature, eliminating the need for electrical power. The detection was performed using a lateral flow strip with a sensitivity of 1.0 TCID 50/mL within 35 min. Qiu et al. [78] used a cellulose membrane integrated PCR device for the detection of *Bacillus cereus*. The device has a limit of detection (LOD) of approximately 10^3^ target cells. The cellulose membrane is used for mechanical lysis and nucleic acid filtration. Khodakov et al. [9] designed special PCR primers to eliminate the need for preliminary purification stages. The special primers contain the main primer sequence and a “tag sequence” that is additionally linked to the main primers through a poly(ethylene glycol) molecule. These special primers are used to generate double standard (ds-PCR) amplification. This primer system allows the hybridization of unpurified ds-PCR products with the capture probes immobilized on a PDMS surface. Manage et al. [110] eliminated the need for DNA extraction from whole blood and enabled amplification of three genomic targets, namely human platelet antigen 1 (HPA1), fibroblast growth factor receptor 2 (FGFR2), and BK virus (BKV), using a commercially available Phusion polymerase enzyme.

Despite the benefit of portability that microfluidics technology brings, there are also specific impediments that must be carefully addressed. Specifically, the efficiency of RT-PCR is reportedly affected by the interactions between the biomolecules and channel surfaces of the device [22], and this is exacerbated by the small size and increased surface-to-volume ratio of POC implementations. Proper passivation of the inner surfaces of any device is a prerequisite for successful microfluidic RT-PCR. Mixing bovine serum albumin (BSA) as a coating reagent into the PCR solution inhibits protein and cell adsorption on both hydrophilic and hydrophobic surfaces. Mixing BSA and Tween minimizes the adsorption of DNA and Taq polymerase onto the surfaces. With the optimal concentration, PCR efficiency has been doubled [22]. Hilton et al. [111] used a Parylene C coating on microchamber surfaces. 

Multiplex PCR was established to amplify multiple targeted genes simultaneously. However, multiplexing is limited with the formation of primer–dimers that occurs due to the amplification of one target sequence over another and through primer–primer interaction. To minimize these interventions, solid-phase PCR was established by embedding one or both of the PCR primers on a solid surface, while other PCR components reside in the liquid phase. A solid phase microchamber PCR chip was developed by Sun et al. [112] for amplification of avian influenza viral RNA. The solid phase is used instead of liquid-based amplification, which allows a 10-fold improvement in amplification. The sample volume is also reduced by a factor of 10 times by incorporating microarrays into the amplification chamber. This amplification process is completed within an hour.

Intelligent primer design also continues to yield improvements in sensitivity, specificity, and detection robustness, which will be critical for multiplexed detection so as to avoid the formation of primer–dimers. With a clever genomic alignment-based primer design to avoid false priming, Wang et al. [113] demonstrated simultaneous detection of seven different mosquito-transmitted zoonotic encephalomyelitis viruses with an analytical sensitivity of 10^2^ copies/μL. Dean et al. [114] developed a multiplexed microfluidic PCR assay for *Chlamydia trachomatis* (Ct). The assay may simultaneously assess nine Ct loci in 20 min using a 33-cycle protocol. The sensitivity and specificity of the multiplexed device were 91.5% and 100%. 

#### 4.1.2. Continuous-Flow PCR

In continuous-flow-based PCR (CF-PCR) amplification, the nucleic acids and reagents flow to stationary heaters to speed up the processing time. The capillary transient-based CF-PCR concept was presented by Nakano et al. in 1994 [115], and an early design by Kopp et al. [116] comprising a serpentine channel CF-PCR with three thermostable copper blocks is widely used in CF-PCR. Flow-controlled pumps regulate the processing duration of every step in the amplification cycle and various optimizations have been invented to speed up the amplification time. This type of pressure-driven flow consumes energy (2–4 times more than stationary PCR [100]) and lacks portability due to the need for an external energy source and precise pumps. For instance, Aboud et al. [117] used high-speed commercial thermal cyclers (SpeedCycler2 and Philisa). They performed direct multiplexed STR amplification from a paper punch within 15 min 54 s. Byung et al. [118] used leaky surface acoustic wave (SAW) devices to generate and distribute heat within the microfluidic amplification chamber. Brunklaus et al. [119] developed an ultrafast microfluidic PCR module (30 PCR cycles in 6 min) by rapidly moving the fluidic plug backwards and forwards using an air pressure mechanism with a syringe pump in a closed reservoir. Nie et al. [120] combined uniform resistors and gradually changed resistors with microheaters to achieve a thermal gradient for PCR to achieve different annealing temperatures. Chun et al. [121] integrated a continuous-flow reverse transcription (RT-PCR) microfluidic system and online fluorescence analysis for the identification of rotavirus. The temperature zones are arranged circularly. However, the RNA detection limit is low—6.4 × 10^4^ copies μL^−1^ compared to conventional immune electron microscopy (>10^4^–10^5^/mL). 

**Figure 8:**
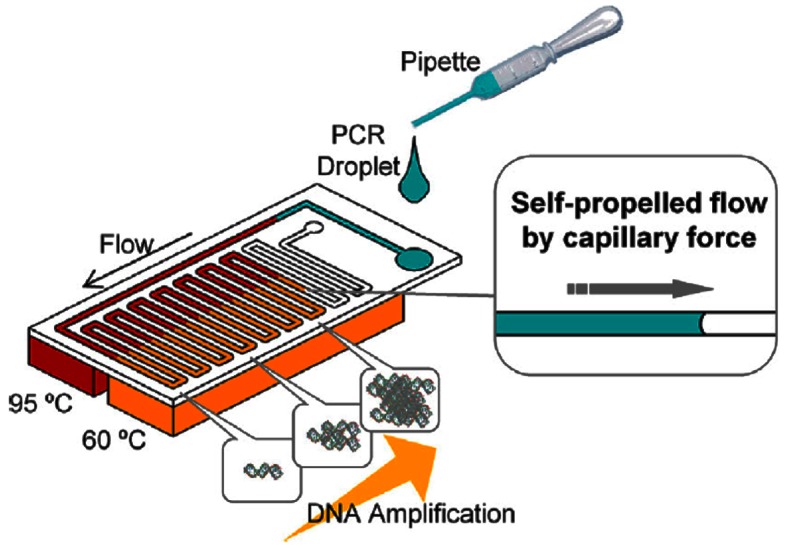
Schematics of self-propelled CF-PCR microfluidic device. The PCR solution is dropped and automatically transported by capillary forces. Reproduced with permission from [123]. Copyright (2015), with permission from Elsevier.

Designs are evolving towards greater POC device portability by using passive methods to speed up amplification or reduce energy consumption. Fast enzymes have been designed to speed up amplification while retaining sensitivity, like the Taq enzyme, which reduces PCR duration from 2.5 h to under 16 min [116]. Wu et al. [122] developed a novel 3D arrangement on a triangular prism to use only a single heater for CF-PCR. Tachibana et al. [123] used capillary flow to passively propel the genomic solution through the CF-PCR stages (Figure 8). To facilitate capillary flow, the device surfaces were made hydrophilic with a coating of non-ionic surfactants; this enabled amplification to be completed within 14 min [124].

Detection sensitivity from CF-PCR is poor because genomic samples are axially dispersed and dilute during flow within the device channel. Due to repetitive high-speed temperature transitions, the solution may evaporate or bubbles may form. Furthermore, the flow rate requirements at different thermal stages of amplification may differ and flow speed is also constrained by the initial quantity of genomic material. Wu et al. [125] used paraffin oil plugs at both the inlet and outlet to eliminate possible nucleation sites for bubbles and also aid in maintaining a stable flow rate for the sample at various temperature zones. However, the oil must be separated from the reaction mixture post-amplification by centrifugation. 

The sensitivity of PCR can be improved by increasing the analyte concentration by tagging the target DNA samples in the volumes of nanoliters and femtoliters. In this context an amendment to PCR, nested PCR, is used to decrease the non-specific binding of the target due to the amplification of unintended primer binding sites. Nested PCR consists of two sets of primers (outer primers and nested primers). The target sequence undergoes a first round of PCR amplification using outer primers. In the second round, the product of the first round acts as a template for nested primers. Shu et al. [126] demonstrated the nested PCR in a continuous-flow format with a sensitivity of 0.2 copies/μL. 

Zhang et al. [127] reported a multiplex oscillatory flow-based PCR system capable of detecting three foodborne bacterial pathogens. The sensitivity was 3.72 × 10^4^ copies/μL (*S. enterica*), 3.58 × 10^4^ copies/μL (*E. coli* O157: H7), and 1.79 × 10^4^ copies/μL (*L. monocytogenes*), respectively. Although continuous-flow PCR offers the advantage of high-speed amplification, it is limited by the following disadvantages. CF-PCR has low detection sensitivity due to the dilution of samples caused by the effect of axial dispersion in the channel flow-field. The speed of the continuous flow PCR amplification depends on the amount of initial base pairs of the genomic nucleic acid. Another common problem is how to satisfy different amplification requirements by regulating different flow rates of PCR solution into the corresponding temperature zones. 

#### 4.1.3. Multiple Annealing and Looping-Based Amplification Cycles (MALBAC)

MALBAC is the method for uniform, linear, whole-genome amplification. The principle uses a uniform, quasilinear pre-amplification of nucleic acids prior to PCR amplification. Briefly, random primers are hybridized to single standard template DNA. Polymerase is used to generate semiamplicons, which are then melted off and processed to form full amplicons with complementary ends. These full amplicons are arranged in a loop structure and detached from the template pool for the subsequent cycles, leading to linear amplification. To end with, the method switched to PCR exponential amplification of full amplicons. MALBAC provides unique uniformity for accurate detection of both copy number variations and single point mutations of individual cells for maximal sequence coverage.

Yu et al. [30] used the MALBAC technique to perform multiplex, single-cell, whole-genome amplification (WGA). Up to eight single-cell MALBAC reactions are performed in parallel using a single device. The whole WGA process, including cell lysis and a two-step MALBAC process containing pre-amplification and PCR amplification, is completed within 4 h with minimal hands-on time.

### 4.2. Isothermal Amplification

To simplify processing, various new reactions have been invented to amplify genomic material at a single regulated temperature. These isothermal methods include nucleic acid sequence-based amplification (NASBA) [128], loop-mediated isothermal amplification (LAMP) [129], recombinase polymerase amplification (RPA) [130], helicase-dependent amplification (HDA) [131], rolling circle amplification (RCA) [132], primer-generation rolling circle amplification (PG-RCA) [133], strand displacement amplification (SDA) [134], transcription mediated amplification (TMA) [135,136], branched DNA signal amplification (bDNA) [137], and single primer isothermal amplification (SPIA) [138]. The most popular among these isothermal amplifications are NASBA, LAMP, RPA, HDA, and RCA. 

#### 4.2.1. Nucleic Acid Sequence-Based Amplification (NASBA)

NASBA was invented by Compton in 1991 [128] and is formulated from nucleic acid transcription for amplification of mRNA, rRNA, tmRNA, genomic RNA, or single-stranded DNA. The reaction saves time by excluding the reverse transcriptase, and suffers reduced interference from DNA (amplification takes place at 41 °C, which is below the temperature threshold for DNA denaturation). With an initial denaturation step at an elevated temperature, NASBA can be modified to amplify dsDNA [139]. NASBA achieves 10^9^-fold amplification in 90 to 120 min. Reinholt et al. [104] isolated and amplified eukaryotic hsp70 mRNA from *Cryptosporidium parvum* within a single-channel microfluidic device. The channel surfaces are modified with Polyamidoamine (PAMAM) dendrimers to inhibit non-specific binding of proteins and nucleic acids and thereby reduce loss of NASBA enzymes and sample mRNA. Tsaloglou et al. [140] integrated a NASBA-based sub-cellular analysis system for the rbcL gene of the phytoplankton *Karenia brevis*. The rear pipetting method was used to eliminate bubble formation during the amplification process. Limit of quantitation (LOQ), defined as the minimum number of cells noticeable above the positive control, is found to be 10 cells in 2.24 min. NASBA processing is not completely isothermal as the primers require initial annealing, which may compromise the sensitivity of the reaction’s enzymes [139].

#### 4.2.2. Loop-Mediated Isothermal Amplification (LAMP)

LAMP was first developed by Notomi et al. in 2000 [141] and uses four target-specific primers to recognize six distinct sites, situated on each side of the template DNA sequence. Briefly, amplification continues through the reiteration of two kinds of elongation reactions that take place through the loop regions. First, self-elongation of the template from the stem-loop structure shaped at the 3′-terminal, followed by binding and elongation of new primers to the loop region. The amplification proceeds between 60 to 65 °C [142] and 10^9^ copies of the target may be produced in 1 h. LAMP integrates amplification with detection and permits a variety of readout options, which is a key advantage. The presence of the targeted nucleic acids may be visualized by the naked eye either as a change in turbidity or fluorescence dye in the reaction chamber [143] or detected by gel electrophoresis or electrochemical measurements. LAMP detection is more robust against impure samples as compared to PCR [144], which is another advantage for deployment as a POC device in resource-starved environments, which leads to gains in detection sensitivity [145] or simplified sample preparation procedures. Hataoka et al. [129] demonstrated the first microfluidic LAMP chip, while Liu et al. [146] and Tourlousse et al. [147] showed the feasibility of microfluidic-based LAMP amplification with a very small quantity of initial nucleic acid templates. Wang et al. [148] demonstrated a one-step real-time LAMP (RT-LAMP) that achieved 100-fold sensitivity gains as compared to RT-PCR. Borysiak et al. [149] developed an integrated device linking ITP with LAMP. They utilized capillary valves and heat-induced pressure driven flow to transport the ITP purified DNA into an LAMP reservoir. Their assay can reliably detect 10^3^ CFU/mL of *E.Coli* in milk sample, which is of two orders magnitude enhancement over regular tube-based LAMP assays. Rafati et al. [142] used microfluidic capillary tubes to perform LAMP amplification and DNA from *Mycobacterium tuberculosis* is detected within 15 min using turbidimetric detection. It is widely reported in the literature that LAMP suffers from self-priming of oligonucleotides during amplification due to four primers in a reaction assay, which causes false positive results of LAMP that can be mitigated by optimizing the assay conditions (concentrations of primers, magnesium ions, deoxynucleotide, and polymerase) and cut-off time [150]. Unfortunately, liquid reagents are transported and stored in frozen form. To facilitate their deployment in POC settings, LAMP reagents can be stored in dried form with a shelf life of up to 30 days at 56 °C [151]. Chen et al. [152] were able to store reagents within a low-melting-point agarose gel maintained at 4 °C for long-term storage. 

LAMP-based methods show great promise for use in POC devices and improvements to enhance the ease of use and assay robustness. Lee et al. [153] pioneered a direct LAMP technique to eliminate the genomic extraction process by utilizing a PCR buffer capable of lysing bacterial cell membrane and inactivating PCR inhibitors in human whole blood or milk. They demonstrated single-cell detection of *Staphylococcus aureus* and *E. coli* in human whole blood or milk within a 1-h processing time. Recently, Ahmad et al. [154] reported an integrated LAMP with most probable number (MPN). MPN-LAMP is a direct cell-based technique that eliminates sample processing steps and the related loss of nucleic acids. LAMP primers are intended for β-D glucuronidase and glutamate decarboxylase genes of *E. coli* and gelatinase gene of *E. faecalis*. The microfluidic chip has an analytical sensitivity of 10 CFU within 20 min. 

Zhou et al. [155] developed a real-time fluorogenic on-chip LAMP capable of simultaneously identifying 10 pathogenic bacteria in aquatic animals. The multiplexed LAMP amplification is also demonstrated using simple microfluidic capillaries [156]. The sensitivity of the multiplex device was found to be 100 orders of magnitude better than typical PCR and is comparable to the sensitivity of real-time PCR. Chen [152] also demonstrated multiplex detection of four bacterial DNA targets with a detection sensitivity of three copies/μL with agarose gel prestoring.

#### 4.2.3. Recombinase Polymerase Amplification (RPA)

RPA [130] is a rapid amplification technique using a precise combination of proteins and enzymes, namely Recombinase, single-strand binding protein (SSB), and strand-displacing DNA polymerase. Recombinase forms complexes with a pair of primers and oligonucleotides with their homologous sequences in double-standard DNA. At that point, SSB binds to the displaced DNA strand and stabilizes the resulting D loop. Finally, DNA polymerase initiates the amplification process. The RPA reaction occurs between 37 to 41 °C and yields 10^4^-fold amplification; RPA amplifications are completed within 10 min with high specificity. RPA was demonstrated by Piepenburg et al. in 2006 [130] with a probe-based detection method. Tsaloglou et al. [157] demonstrated identification of *Clostridium difficile* with a detection limit of 1000 DNA copies (corresponding to 1 fg) in 20 min with a real-time microfluidic RPA chip. Eid et al. [70] showed RPA detection of *L. monocytogenes* bacteria in whole blood but with a poor LOD of 2 × 10^4^ cells/mL, presumably due to lysing. RPA reagents are stable at 45 °C for up to three weeks [158] and can be transported without freezing; this is a key advantage for portability, especially for use in resource-limited areas [159]. 

Kersting et al. [160] used solid phase RPA amplification to minimize the formation of primer dimers and decrease the amount of non-specific products in a multiplex amplification. They developed on-chip RPA for simultaneous detection of *Neisseria gonorrhoeae*, *Salmonella enterica*, and methicillin-resistant *Staphylococcus aureus* (MRSA). This on-chip RPA is able to amplify 10 copies of genomic DNA, making it a particularly sensitive method. 

Although RPA is a rapid amplification technique, it suffers from unwanted preliminary amplifications at room temperature when initiation reagents are premixed with the nucleic acid sample. The preliminary amplifications result in false positive results, which can be abolished by dividing the nucleic acid template into sections earlier and adding initiation reagents [161,162]. Another drawback of RPA is the interaction between well-designed primers. This can interfere with the amplification and quantization of the sample. This can be circumvented by using a Self-Avoiding Molecular Recognition System (SAMRS). These SAMRS are a nucleotide sequence that will bind only to DNA and not to other nucleotide sequences [162,163]. In addition to this, RPA is inhibited by whole blood [164] and large background DNA [165]. 

#### 4.2.4. Helicase-Dependent Amplification (HDA)

HDA was introduced by Vincent et al. [131] in 2004. The method uses a DNA helicase instead of elevated temperature to denature dsDNA into single-stranded templates, thereby enabling primer-oriented isothermal amplification of templates of up to several kilobases long. Besides the capacity to amplify long nucleic sequences, the operating temperature of HDA is also an advantage to simplify devices. The two primers and DNA polymerase are annealed at 37 °C. Huang used a toe warmer while Kaprou et al. [166] used copper on PCB as a heater for continuous-flow HDA amplification of *Salmonella*. Madhumita et al. [167] used a fluorescent report to detect down to 10 CFU of *E. coli* and Huang et al. [168] detected *Clostridium difficile* with a limit of detection down to 1.25 × 10^−2^ pg. HDA shares common disadvantages with RPA and LAMP in that the DNA in the amplification mixture can easily form non-canonical folds or primer dimers, which may result in low detection sensitivity, and elevated false positive and false negative results. These problems can be alleviated by SAMRS [169] or by blocked-primer helicase-dependent amplification (bpHDA) [170]. In bpHDA, blocked primers are designed with a single ribonucleotide linkage introduced four bases upstream at blocked 3′-end to avoid primer extension. These bpHDA cannot be directly extended; instead, a hot-start using RNase H2 is required, which makes bpHDA unsuitable for RNA targets. 

#### 4.2.5. Rolling Circle Amplification (RCA)

The RCA process amplifies circular nucleic templates by the addition of nucleotides to the template to create long ssDNA with repeated tandems under the influence of DNA or RNA polymerase. Amplification is linear at 37 °C and 10^3^-fold increase can be achieved in 1 h. Hyper-branched RCA (HRCA) (or ramification amplification) is an exponential amplification variant where RCA products are used as a template for further amplification with a second or third set of primers. HRCA requires activation at 60 °C and achieves 10^9^-fold amplification in 90 min. With the aid of the catalyst hemin/G-quadruplex, RCA achieves excellent sensitivity and may detect a single DNA molecule and distinguish between DNA with single base pair resolution. Kuroda et al. [132] demonstrated single DNA molecule counting in HeLa cells on an automated RCA microfluidic system and Lin et al. [171] demonstrated the ultrasensitive detection of 0.083 pg/mL of the protein thrombin for disease detection. However, several disadvantages pose significant barriers to the adoption of RCA as a POC technique. High-purity and high-quality circular templates are required and can be prepared by exonuclease-assisted degradation of a low concentration of linear DNA. RCA products are apt to aggregate due to non-specific binding, which introduces complications in HRCA detection. 

### 4.3. Digital Amplification

Digital amplification of nucleic acids is a novel approach that integrates amplification and detection to generate additional information about the quantity of genomic material present in the sample. The nucleic acid containing solution is separated into many smaller portions as microwells or droplets. In every portion, the presence of the target nucleic acid is assayed by amplification and binary detection. Analyzed together, all volume portions express the probability that the target genomic material is present in a subvolume and the quantity of genomic material initially present can be inferred. Detection sensitivity, accuracy, and resolution are strongly dependent on the total number and volume of portions. Ideally, each microwell/droplet should accommodate only one or zero copies of the target sequence and the amplification technique should be sufficiently sensitive to detect single copies. Preliminary applications of quantitative genomic information have been applied in studies of diseases with a strong genomic component like breast cancer to measure disease-related variations in gene expression, copy number variations, or allelic imbalance of genes. We believe there is potential for new applications in the field of clinical diagnostics. 

To identify which amplification technology was best suited for quantification of genomic material, Nixon et al. [172] compared the performance of quantitative PCR (qPCR), digital PCR (dPCR), quantitative LAMP (qLAMP), and digital LAMP (dLAMP) on human cytomegalovirus (hCMV). dPCR was more sensitive than dLAMP and direct dPCR was more sensitive than qPCR. dLAMP outperformed qLAMP and was less impeded by amplification inhibitors. Their results imply that digital amplification supersedes qPCR and qLAMP in the task of quantifying genomic content. 

Microfluidic innovations around digital amplification are thus far confined to methods for subdividing the fluidic sample and to amplification for binary detection. The sample solution can be separated into droplets or volume-controlled microwells. Both PCR and LAMP amplification have been applied, typically with fluorescence readout [173,174,175]. In microfluidics, droplets are generated either through active systems (controlling valve—piezoelectric valve) or passive systems (pressure driven nozzles or T-junction) [176]. Droplets are formed in these systems by emulsions of oil, water, and other stabilizing chemicals. Hatch et al. [177] developed an ultrahigh-throughput ddPCR, whereby 1 million droplets are processed and analyzed in real time. In this device, 50 μL of PCR reaction is discretized into 1 million 50 pL droplets using a 256 droplet-splitter design. The incorporation of high-throughput increased the dynamic range of ddPCR by 100-fold. However, droplet uniformity, overlapping, and coalescence hamper real-time monitoring in this device. 

Droplet-based digital amplification needs uniform droplet formation for accurate DNA quantification. The average volume of droplet changes with the viscosity of DNA molecules [178] and also with the droplet generator. For viscous DNA molecules, shearing and fragmentation of DNA are recommended to guarantee uniform droplet size [178]. Towards droplet generation, Bauer et al. [179] coated certain regions of the PDMS microchannel with polyelectrolytes to create partially hydrophilic and partially hydrophobic regions to generate uniform droplet emulsions. The standard deviation is less than 1% for a 35.8-μm droplet. Sang et al. [180] used liquid–gas phase transition to generate uniform submicron-sized droplets. The principle is based on the solubility of the gas. Two gases with different solubility (soluble in water and insoluble) are mixed with aqueous surfactant solution using a microfluidic device to generate uniform droplets. The soluble gas diffuses out of the solution while the insoluble gas condenses into droplets. The standard deviation is about 8% for a 0.7-μm droplet. In addition, oil and chemical emulsions may be absorbed by PDMS surfaces during transport to the reaction chamber, resulting in loss of sample encapsulation. Bian et al. [181] saturated the PDMS substrate of the microfluidic chip with mineral oil (OSP), which prevented the droplets from evaporating during PCR thermal cycling and was successful in detecting *E. coli* and *L. monocytogenes* and quantification of microRNA in lung cancer [182]. 

Rane et al. [174] developed a system with a continuous flow of droplets through a wide serpentine channel. LAMP reagents were mixed with the sample before digitization into picoliter-sized droplets and, using fluorescence readout, 10 μL of sample could be processed in 110 min. Schuler et al. [183] generated droplets by centrifugal step emulsification, performed LAMP amplification in an in situ cycler, and used fluorescence image detection with a standard microarray scanner. They achieved absolute quantification of DNA in the range of 15–1500 cp/mL. Schuler et al. [184] demonstrated first centrifugal digital droplet RPA (ddRPA) using a polymer cartridge. The total analysis time was 30 min. However, ddRPA performance was affected by bulk amplification, which is controlled by fast droplet generation. Moreover, RPA cannot be initiated through hot-start to avoid bulk amplification. Droplet technology is not readily translated to POC applications because portable and cost-efficient micro-droplet generator and droplet readout systems have not yet been invented. Furthermore, the reagents for amplification must be optimized for emulsions. 

Microwell technology is more amenable to POC deployment, though not in resource-limited settings. In the simplest approach, the fluid sample is equally divided and transported through a network of microchannels to an array of microwells [185,186]. Each microwell has the same volume, which is advantageous for detection accuracy, but any excess sample volume exceeding the combined well capacity are flushed out, which can be a significant loss for applications involving rare sample detection. Ismagilov et al. [187,188] developed the SlipChip, a microfluidic device to perform multiplexed microfluidic reactions. The device consists of two plates, a bottom plate and a top plate. The bottom plate contains ducts the top plate has microwells. The top plate is “slipped” against the bottom plate in a specific configuration to induce fluidic connections and enable mixing of the sample in the top plate with the preloaded reagents in the bottom plate. The performance of SlipChip is limited in terms of the number of microwells and in realizing perfect alignment of plates with 100 μm microwell size. Several researchers have tried to harness viscoelastic fluid phenomena to initiate “self-digitization” (SD) of the fluid sample. Schneider et al. [185] discovered that very stringent control of the device geometry is required. Thompson et al. [186] succeeded in using the SD chip for quantification of mRNA from single cells using RT-PCR. Later Zhu et al. [189] developed self-priming compartmentalization (SPC), which eliminates the need of valves and pumps for discretization. The energy for pumping is realized by exploiting the PDMS gas solubility property by degassing bulk PDMS. On release, the pressure difference of gas dissolved in PDMS sucks the sample and solution in, resulting in self-compartmentalization. 

**Figure 9:**
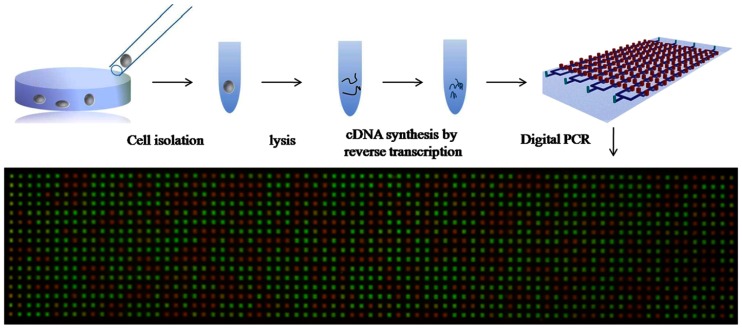
Self-priming compartmentalization (SPC) digital PCR chip for single-cell gene expression. Reprinted from [191], with the permission of AIP Publishing.

Zhu et al. used SPC with PCR to detect lung cancer genes [190] and perform quantification of single cell gene expression [191]. SPC chips do not use complex geometries or flow control. Fu et al. [192] added surfactants in pre-cured PDMS to inhibit the absorption of biological macromolecules on PDMS, which would interfere with the reproducibility of PCR results. Zhu et al. [191] introduced fractal tree-like microchannel nets, which enable the entire sample solution to be digitized and analyzed. The process of SPC digital PCR is shown in Figure 9. Yeh et al. [193] demonstrated digital nucleic acid amplification using RPA. The device utilizes self-powered microfluidic pumping to separate plasma from blood and to dispense the sample and reagents into 224 microwells. In this work, the RPA amplification initiator (magnesium acetate (MgOAc)) is prepatterned on the chip and separated from the master mixture to avoid bulk amplification. 

Multiplexing of ddPCR in the same assay is achieved by coupling distinct fluorophores or through coupling encoded bead-based array or Luminex suspension array. The usage of fluorophores and encoded bead-based array for multiplexing is limited to five targets in the same assay due to spectral overlap. In contrast, Luminex beads are distinguishable in a wide spectral range due to their red and infrared dye composition. Zonta et al. [194] multiplexed ddPCR for cancer-related mutations using fluorophores and Rajeswari et al. [195] used Luminex beads for multiplexed detection of avian influenza, infectious laryngotracheitis virus, and *Campylobacter jejuni*. Liu et al. [196] reported a LAMP-based microfluidic system for parallel analysis of *Mycobacterium tuberculosis*, and magnetic beads are coupled with droplet technology for detection in polytetrafluoroethylene capillaries. During the process, different types of samples or reagents are successively introduced in the form of liquid plugs and droplets are generated within capillaries. The system was able to process 10 samples in parallel within 50 min and with an LOD of 10 bacteria. This microsystem has a sensitivity of 96.8% and specificity of 100%. 

## 5. Detection

At completion of processing, a POC diagnostic device must make available the results of genomic identification, usually as a binary decision or quantitative value of the genomic content. Conventionally, this information is conveyed by visualizing a change within the reaction chamber (commonly a change in color) or by electrochemical measurements. The amplification and detection steps are often integrated and the readout could occur at the end-point of processing or throughout the amplification step as a real-time signal. Fluorescence detection is the most widely used technique in microfluidics because of high selectivity, sensitivity, and efficiency [197]. A fluorescence indicator binds to the target nucleic sequence and emits light of a pre-designed wavelength in response to a laser or Light-Emitting Diode (LED) excitation. The concentration of targeted nucleic acids can be obtained from the intensity of the emitted light [197]. 

There are various reports of device innovations to improve the device portability and cost. Researchers have miniaturized optical detection methods from microscopy [112,198,199] and integrated with novel light sources like organic LEDs, filter-free photodiodes [200], and waveguides [201] to enhance the signal intensity and assay sensitivity, primarily in PCR-based systems. Hung et al. [202] developed supercritical angle fluorescence (SAF) detection with a microlens array for use with solid-phase PCR. Tae et al. [203] integrated an RT-PCR microdevice and an immunochromatographic strip for colorimetric detection of influenza A virus subtype H1N1. Colorimetric detection (violet color), as a resultant of gold nanoparticle (Au-NP), is used to confirm the presence of H1N1 target virus. Texas red primers and biotin-labeled nucleotides are used in amplification. The resultant amplicons are conjugated with gold nanoparticles labeled with an antibody. 

There are challenges to the robust deployment of fluorescence indicators in POC devices because the indicators are costly, sensitive to pH and temperature, require sensitive detectors and collection optics, suffer interference from sample autofluorescence, and require storage at low temperatures. In LAMP-based systems, the amplification reaction precipitates out magnesium pyrophosphate, which makes the solution turbid [204]. Microfluidic chips can detect the changes by measuring light intensity reflected from the solution [205]. Lin et al. [206] used optical fibers for light delivery and collection to reduce the variability of the signal from dust, dirt and manufacturing variability of the device. Safavieh et al. [204] employed hydroxy naphthol blue (HNB) and calcein dyes for colorimetric detection of positive LAMP reactions. The device is able to detect *E. coli* and *S. aureus* in less than 1 h with microfluidic cassette. The cassette contains 36 chambers for simultaneous analysis. LAMP reagents are more robust to deployment in POC settings.

**Figure 10:**
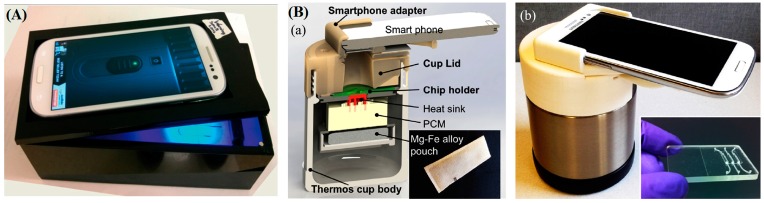
(**A**) NAIL mobile imaging unit consisting of a dark box with an excitation and emission filter, and mobile phone. Reproduced from [149] with permission of The Royal Society of Chemistry. (**B**) (**a**) Exploded view of smart cup. Inset is a snapshot of the Mg–Fe alloy pouch used as a heating source. (**b**) A photograph of a fully assembled smart cup. Inset is a snapshot of an integrated microfluidic chip. Reprinted from [209]. Copyright (2016), with permission from Elsevier.

Smartphones are supplanting microscopic detection as a popular platform for optical detection in POC settings. Widely available, portable and low-cost, smartphones come with embedded sensitive imagers and optics with 24-bit color resolution and Bayer filters for color detection [207] and powerful software to manage the image acquisition and processing. Many groups have developed minor optical extensions to commercial smartphones like adding filters and optical fiber assemblies to enable fluorescence detection of LAMP [149,208,209] and PCR [199] microfluidic systems (Figure 10). Power-free LAMP amplification and smartphone detection was demonstrated by Liao et al. [209]. The device is named a smart cup, as shown in Figure 10B. The temperature required for amplification is generated using an exothermic chemical reaction between magnesium and water in the presence of iron. 

**Figure 11:**
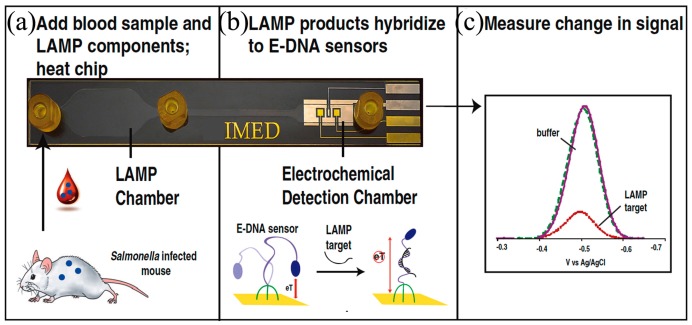
Electrochemical detection: (**a**) Unprocessed, whole blood from infected animals is introduced into the chips. The reaction mixture containing single-stranded amplicons is then pushed into (**b**) the electrochemical detection chamber. This chamber contains a duplexed electrode array that supports simultaneous, sequence-specific electrochemical detection. (**c**) Generating a detectable decrease in current. Reproduced from [210] with permission from American Society for Microbiology.

Electrochemical detection employs electrodes that directly interface with the sample solution to measure changes in sample conductivity due to ongoing reactions and byproducts. Microelectrodes or screen-printed electrodes offer the advantages of low cost, disposability, and not interfering with the reactions within the sample. Detection is reagent-free, sequence-specific, and amenable to multiplexed sensing array, which are important advantages for POC devices. Patterson et al. [210] integrated a microfluidic LAMP with an electrochemical DNA (IMED) chip for the detection of *Salmonella enterica*. The microfluidic chip contains an amplification chamber and a detection chamber, as shown in Figure 11. The detection chamber consists of a sequence-specific, multiplexed (here a biplex) sensing array for electrochemical detection of amplified products. The device has a LOD of less than 10^3^ CFU/mL.

Some challenges still hinder robust deployment in POC, namely the fact that signal measurements are highly dependent on variations in temperature, pH, and ionic concentrations, thus limiting measurement sensitivity. Electrodes measure the reduction in redox current as the concentration of DNA-binding methylene blue in the reaction drops due to amplification of DNA in LAMP [211], RCA [212], and HAD [213] systems. Redox active compounds may have undesirable interactions with dsDNA or ssDNA and interfere with readout accuracy. To circumvent this problem, Ahmed et al. [214] used a RuHex redox molecule to bind double-stranded amplicons without inhibiting LAMP amplification. Safavieh et al. [215] utilized a redox-reactive osmium complex instead. Luo et al. [216] multiplexed LAMP along with electrochemical detection and reported it as μME-LAMP. The device is able to simultaneously detect three pathogenic bacteria. Independent chambers are utilized to measure the redox current. 

## 6. Novel Microfluidic Actuators

PDMS elastomer and thermoplastic polymers like PMMA, polycarbonate, and cyclic olefin copolymer (COC) have been the preferred material for microfluidic device construction for many years because of the ease of fabricating complex channel geometries and microstructures with these materials and the ease of visualizing the processing and results due to the optical transparency of the polymers. Device designs using these polymers require external pumping mechanisms to move fluids through a network of channels, frequently under controlled flow rates. The pumps and fluidic control impose restrictions on lowering the cost or portability of these microfluidic devices. 

New fluidic actuation mechanisms have recently been invented; we discuss three important advancements here. Cellulose paper or similar cloth and paper sheets provide a passive mechanism for moving fluid simples because the porosity and hydrophilicity of the surface generate capillary flow without the need for external pumps. Centrifugal microfluidics simplify the motorized actuation of flow by utilizing centrifugal forces to transport the sample and reagents within microfluidic channels. Pumps can be replaced by simple motors or manual methods of rotation. Digital microfluidics (DMF) also eliminate the need for pumps because discrete droplets can be precisely repositioned by programmatically manipulating electric or magnetic fields. DMF provides reconfigurability and greater precision in the spatial and temporal manipulation of fluidic samples. 

### 6.1. Paper-Based Microfluidics

Paper-based microfluidics were introduced by Whitesides et al. in 2007 [217] and act as an alternative system for fluid handling with unique and distinct advantages as a substrate for POC diagnostic devices. The paper substrate is foldable and extremely portable, easy to manufacture, and the fluid is passively actuated on the paper surface by capillary forces. Whitesides et al. [218] created a variety of 2D and even 3D microfluidic channels on paper to transport liquids along prescribed routes. Paper microfluidic technology has evolved to sufficient maturity for POC diagnostic devices. The lysis, extraction, amplification, and detection processes can be implemented in paper microfluidic devices. Furthermore, unlike PDMS in conventional microfluidic fabrication, paper is biocompatible and non-toxic to cells. However, the paper substrate introduces new challenges in sample retention and loss due to evaporation as well as poor detection limit and ambiguity in identifying the colorimetric results. 

Fronczek et al. [219] demonstrated chemical lysis, elution with Tris-EDTA (TE) buffer, and smartphone detection to extract *S. typhimurium* with a 5-min processing time. Li et al. [220] demonstrated paper-based ITP for DNA. The device was constructed using origami techniques—the traditional art of Japanese paper-folding—and operated by two 9 V batteries. Moghadam et al. [221] also demonstrated ITP on a nitro-cellulose paper device. They devised cross-shaped design to minimize Joule heating and sample evaporation by hydrating the paper. The extraction was carried out on volumes up to 100 μL with a 900-fold increase in concentration with efficiencies between 60–80%. Rohrman et al. [159] demonstrated amplification of 10 copies of HIV DNA within 15 min using RPA with a multilayer device of paper, glass fiber, and plastic. 

**Figure 12:**
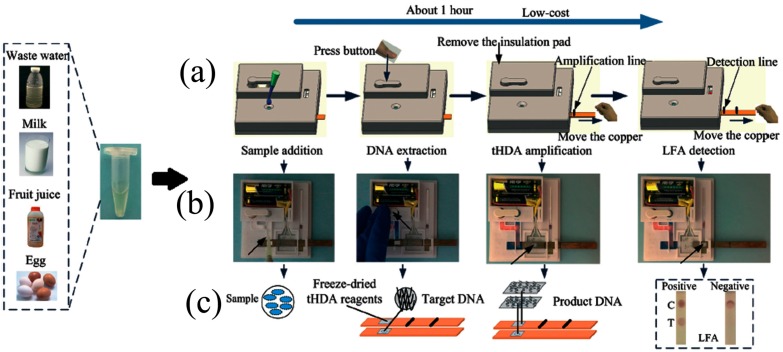
Fully disposable and integrated paper-based sample-to-answer device. (**a**) Schematic diagram of the operations; (**b**) different steps in the operational module; (**c**) schematic diagram of the important operations. Reproduced from [223] with permission of The Royal Society of Chemistry.

Several researchers have recently reported complete POC systems that are ready for pathogen detection. Rodriguez et al. [16] successfully demonstrated chaotropic lysis, DNA extraction through filtration, RT-LAMP amplification, and detection of Influenza A (H1N1) using three separate modules on poly(ether sulfone) filter paper. The device has a detection limit of 10^6^ copies/mL with a total sample—to answer the assay time of 45 min. Later, the same group integrated all three modules onto a modular and foldable paper microfluidic chip for the detection of human papillomavirus [150]. However, there is a risk of sample contamination due to the manual folding and ripping process. Connelly et al. [222] reported a “Paper machine” that chains paper-based extraction, amplification, and detection steps using a magnetic sliding strip to execute each processing step in sequence. Recently, Tang et al. [223] demonstrated a disposable, integrated paper-based device with on-chip dried reagents. The device incorporates HDA and LFA for naked eye detection of *S. typhimurium* with a detection limit of 10^2^ CFU/mL in wastewater and egg, and 10^3^ CFU/mL in milk and juice in about an hour (Figure 12).

### 6.2. Centrifugal-Based Microfluidics

Centrifugal microfluidics or Lab-on-CD utilizes centrifugal forces from a rotating compact disc (CD) for fluid control in microfluidic channels. Centrifugal forces eliminate the need for external pumps for fluidic control. The radial symmetry of the CD allows parallel processing channels for different biological fluids on a single CD [224]. Recent advances in designs for valving, mixing, aliquoting, and pumping have made the Lab-on-CD a promising technique for POC genomic detection [225].

Researchers have reported a variety of designs to perform extraction, amplification, and detection from lysates. Jung et al. [226] developed bead-based RNA purification and real-time optical detection for the influenza A H1N1 virus. The lysate is prepared off-chip by chemical lysis and amplified on the CD using RT-LAMP method. The device required 7 min for RNA purification and 40 min for RT-LAMP amplification with LOD of 10 copies. Felipe et al. [227] proposed an in-disc loop-mediated isothermal amplification (iD-LAMP) and RPA on semi-automated DVD [228] with the capability to analyze 96 samples simultaneously with a sensitivity of 5 CFU/mL and 10 mg/g of bacteria in meat within 15 min. Sayad et al. [229] also demonstrated automated pumping, mixing, metering, and sealing for LAMP amplification on disc for detection of *Salmonella enteritidis* with 70 min total operation time and detection limit of 5 × 10^−3^ ng/μL with off-disk lysis.

**Figure 13:**
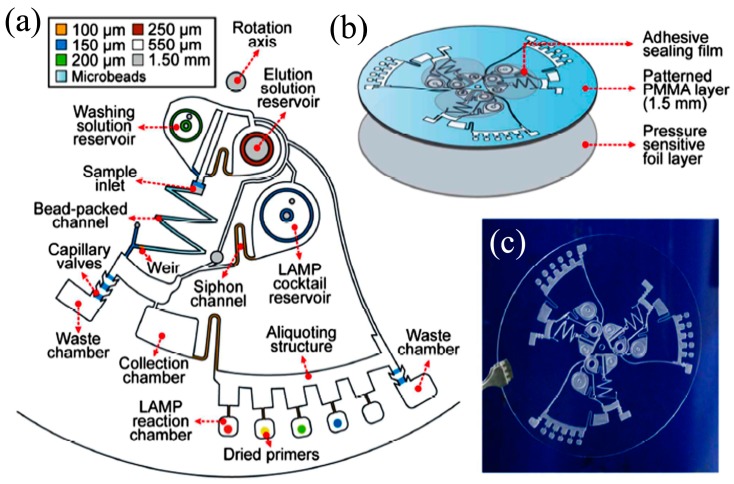
Lab on disc: (**a**) Schematic of lab on a disc with DNA extraction and purification using beads packed inside a microchannel, LAMP reservoir for multiplexed amplification; (**b**) assembly of the disc with patterned PMMA layer; (**c**) fully assembled lab-on-a-disc. Reproduced from [232] with permission of The Royal Society of Chemistry.

Designs capable of multiplex detections are also becoming common. Strohmeier et al. [36] automated the extraction of nucleic acids of *E. coli*, *B. subtilis*, and Rift Valley fever virus from whole blood. They integrated chemical lysis and magnetic bead-based extraction on a disk with total lysis and extraction time of 30 min. Nucleic acid recovery from each pathogen was between 58.2–98.5%, 45.3–102.1%, and 29.5–34.2%, respectively. Oh et al. [230] implemented centrifugal LAMP for simultaneous genetic analysis of 25 pathogen samples by optimizing zig-zag channels and speed control for sequential loading of reagents. Lysis and extraction of nucleic acids are carried out off-CD. On-CD amplification is completed within 60 min with a detection limit of 380 copies. Chang et al. [231] developed a similar multiplex LAMP system for the detection of hepatitis B (HBV), hepatitis C (HCV), and cytomegalo virus (CMV) utilizing Coriolis force for flow control. Oh et al. [232] developed the LAMP-based device shown in Figure 13 for multiplexed detection of four types of foodborne pathogens in milk samples. Naked eye detection is possible with LOD of 10 bacterial cells for *E. coli* O157:H7. Choi et al. [233] also developed a direct-RPA system for multiplexed and real-time identification of *Salmonella enterica*, *E. coli* O157: H7, and *Vibrio parahaemolyticus* in milk samples. Seo et al. [234] modified this system to incorporate multiplexed LAMP detection of the three pathogenic bacteria in 24 reaction chambers on a single device with detection sensitivity of 500 copy level and 100 cell level. Park et al. [235] reported another Lab-on-disc system capable of multiplex operation using glass microbead-based DNA extraction with LAMP and colorimetric lateral flow strip detection of bacterial samples. 

Most Lab-on-disc designs still lack an integrated lysis and nucleic acid extraction step, which is the crucial final piece needed to enable portable and full POC functionality. Another major difficulty is the design and fabrication of valves that function under centrifugation. However, Lab-on-disc technology is rapidly maturing towards fully integrated CD devices. Kim et al. [236] developed an integrated device for *Salmonella* detection using a single laser diode for thermal lysis, valve actuation and non-contact heating during isothermal RPA amplification. Process was completed within 30 min with a detection limit of 10 CFU/mL in Phosphate buffered saline (PBS) and 10^2^ CFU/mL in milk. We anticipate many more fully integrated and multiplexed solutions to be available soon.

### 6.3. Digital Microfluidics

Digital Microfluidics (DMF) devices manipulate fluids by actuating droplets through electric or magnetic field induced motion. Instead of flow inside channels, fluid droplets are placed above an array of electrodes or a network of permanent magnets or electromagnets and the distribution of electric or magnetic fields are controlled to reposition, mix, or dispense droplets. DMF has been a powerful platform for biological applications towards reagent mixing, splitting, transport, and dispensing. 

**Figure 14:**
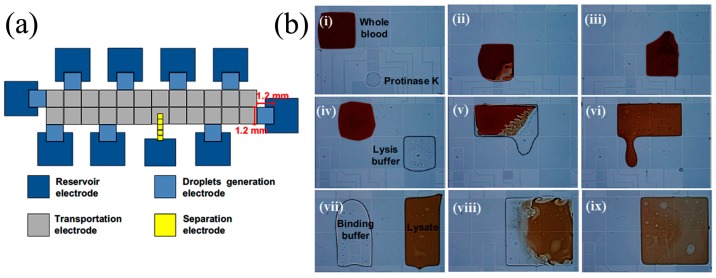
Digital microfluidic chip for DNA extraction. (**a**) Schematic of DMF chip; (**b**) Extraction process: (**i**–**iii**) degradation of nucleases using proteinase K; (**iv**–**vi**) lysis buffer was added to the droplet; (**vii,viii**) binding of DNA with beads; (**ix**) the mixture was mixed in a loop motion to obtain a uniform droplet color. Reproduced from [237] with permission of Springer.

Electric field actuation uses the principle of electrowetting-on-dielectric (EWOD), where electric fields are used to modify the wettability of droplets on a dielectric solid surface. The electric field is generated by patterned electrodes within a dielectric layer and the droplets are positioned on a hydrophobic layer that is fabricated above the dielectric-electrode plane. The 2D array of electrodes is fabricated on ITO glass, printed circuit board (PCB), or paper by inkjet printing. Hung et al. [237] demonstrated a magnetic-bead-based DNA extraction protocol from whole blood on a DMF chip. The extraction process is shown in Figure 14. A pair of droplet manipulation policies was established for cell membrane lysis, binding of magnetic beads, and washing and elution procedures. Rival et al. [238] developed an EWOD chip to chemically lyse, extract (using magnetic beads), and amplify mRNA using RT-PCR on a single or a few cells within a droplet in order to prepare cDNA for deep sequencing. Kalsi et al. [239] reported an EWOD device capable of detecting multiple genes using an RPA triplex assay. The device employs electric field control to automatically dispense DNA and reagents simultaneously on 45 droplets and achieved a LOD of 10 copies in 25 min. EWOD devices require precise patterning of electrodes for droplet transport and the tolerances of EWOD are quite low within the expected range of accuracy [240]. 

Magnetic field actuation utilizes magnetic beads as the substrate to actuate fluid droplets (magnetic beads within the fluid droplet move under the influence of the magnetic field and the fluid is carried along with the beads because of surface adhesion of the fluids onto the large surface area of the beads) and for adsorption of nucleic acids. Zhang et al. [241] reported a droplet microfluidic device for the detection of infectious pathogens from human blood and bacterial culture. The droplets are actuated using a permanent magnet and integrate chemical lysis, magnetic-bead-based extraction, real-time PCR, and fluorescence detection. The device also incorporates surface topographic features (slits) to increase the splitting efficiency of magnetic particles from the droplets. The usage of permanent magnets limits the strategies for bead actuation, resulting in an inefficient extraction process. Moreover, permanent magnets require bulky translational stages for automation of diagnostic procedure. Later the same group proposed electromagnetic droplet platform using an array of planar coils for droplet actuation [242]. The same group also identified genetic mutations [243]. 

Several restrictions must be overcome before DMF technology is ready for deployment in POC devices. Since fluid droplets are actuated on an exposed surface, rapid evaporation of the fluid is a major challenge. At present, the droplets are encapsulated with mineral oil during the amplification process to minimize evaporation and reduce voltage in EWOD. However, the use of oil significantly slows down the thermal ramping rate and prolongs the processing time. Cross-contamination and the need for proper cleaning procedures also limit the reusability of DMF devices.

## 7. Multiplex Integrated Devices

Despite the large variety of methods for lysis, extraction, amplification, and detection, microfluidic devices for POC testing are still not prevalent. We anticipate that the integration of these techniques into an on-site diagnostic device with rapid processing time, simultaneous testing for a range of pathogens (multiplexed detection), and sufficiently sensitive detection limit will lead to a new generation of POC diagnostic devices. Multi-target diagnosis can be performed using multiplex amplification such as multiplex PCR, Multiplex LAMP, Multiplex HDA, and Multiplex RCA. In multiplex amplification, several target DNA sequences are amplified simultaneously by utilizing multiple primers in a single reaction microchannel/chamber. However, multiplex amplification needs careful primer design to eliminate primer dimers as well as optimization of other components in the master mix. Multi-target diagnosis can also be achieved by using several parallel detection channels on the device, where each channel tests for a different type of pathogen. 

The system developed by Wang et al. [244] was used to distinguish between infA/H1, infA/H3, and infB. Their device utilizes magnetic beads to capture, thermally lyse, and extract viral RNA or multiplexed RT-PCR with fluorescent readout. The LOD of multiplex detection was 10^2^ copies with ~110 min processing time. Later Tai et al. [245] of the same group established parallel amplification in an integrated microfluidic platform using separate chambers for RT-PCR. The platform achieved a sensitivity of 90% and a specificity of 100% within 60 min. 

**Figure 15:**
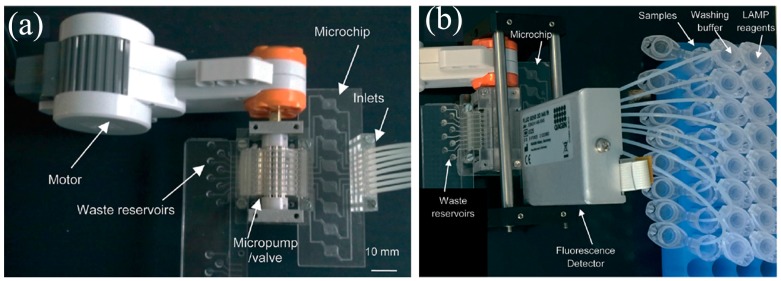
(**a**) Integration MainSTREAM micropump with microchip; (**b**) an eight-chamber microfluidic chip was connected with eight-channel peristaltic micropump to load the sample and reagents, integrated with a fluorescence detector. Reproduced from [246] with permission of The Royal Society of Chemistry.

Sun et al. [246] integrated the magnetic-bead-based extraction, LAMP amplification, and real-time detection of *Salmonella* in a single chamber. The chip contains eight chambers for parallel reactions and has a detection limit of 50 cells per test with a detection time of 40 min. Sample and magnetic beads were mixed with a lysis buffer and transferred to the microchip. A multi-channel pumping was used to transport washing buffers and LAMP reagents. However, the use of multiple reagents washing steps and fluorescence detector makes the system cumbersome to use as a POC device, as shown in Figure 15. Branavan et al. [247] developed an integrated microfluidic chip. The chip performs chemical lysis, membrane filtration, and HDA or RPA amplification. A low-cost amplification platform can perform six amplifications simultaneously. The chip has a detection limit of 1.32 × 10^6^ of sample DNA through HDA and 1 × 10^5^ copy numbers for *Chlamydia trachomatis* genomic DNA within 10 min through RPA. 

Recently, Dou et al. [248] developed a polymer/paper hybrid microfluidic biochip for multiplexed detection of *Neisseria meningitidis* (*N. meningitidis*), *Streptococcus pneumoniae* (*S. pneumoniae*), and *Haemophilus influenzae* type b (Hib). The biochip contains eight parallel LAMP zones for amplification and detection. The paper functions as a substrate for DNA primers. The hybrid microfluidic biochip was found to have a shelf life of three months compared to other microfluidic chips. LOD is found to be comparable to or higher than other reported qPCR techniques. LOD is found to be 29.6 fg/μL for N. meningitides, 56.1 fg/μL for *S. pneumonia* and 39.7 fg/μL for Hib. Bian et al. [181] demonstrate the duplex droplet digital PCR (ddPCR) for simultaneous detection of *E. coli* O157 and *Listeria monocytogenes*. They employed two fluorescent probes (two-color TaqMan-MGB) to detect both bacteria simultaneously with LOD of 10 CFU/mL and 2 h processing time. 

**Figure 16:**
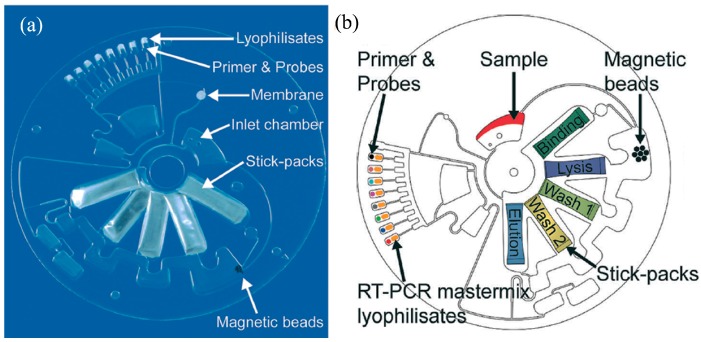
(**a**) Photograph of the LabDisk for respiratory pathogens with complete reagent prestorage; (**b**) schematic of device with sample supplied into the LabDisk—automated to pump the sample into the lysis chamber by centrifugation and therein with prestored magnetic beads. Finally, reverse transcription and PCR with real-time fluorescent readout are performed. Reproduced from [250] with permission of The Royal Society of Chemistry.

Czilwik et al. [249] demonstrated a centrifugal disk platform for identification of *Staphylococcus warneri*, *Streptococcus agalactiae*, *E. coli*, and *Haemophilus influenzae* from a blood serum sample. The disk integrates chemical lysis, silica-coated magnetic-bead-based extraction, nested PCR amplification, and fluorescent detection. The eluted DNA is pre-amplified and subsequently divided into 13 reaction chambers for target specific amplification. The device has a LOD of 15 CFU/mL of *Staphylococcus warneri*, 10^3^ CFU/mL of *Streptococcus agalactiae*, 25 CFU/mL of *E. coli*, and 10 CFU/mL of *Haemophilus influenzae* within a processing time of 3 h and 45 min. The same group (Stumpf et al. [250]) presented a fully integrated centrifugal LabDisk with complete pre-storage of essential reagents for the detection of influenza A H3N2 virus (Figure 16). The total time for the sample to result is 3.5 h with a detection limit of 75 PFU/mL. Roy et al. [251] demonstrated a robust, integrated, microfluidic Lab on a CD fabricated from a low-cost thermoplastic elastomer. The disk integrates mechanical lysis, droplet mixing, PCR amplification, and amplicon digestion and hybridization onto a microarray. The device allows four parallel multiplexed reactions. However, a 15-step process is needed for the identification of *Bacillus atrophaeus* subsp. globigii spores. 

## 8. Commercial Microfluidic Diagnosis 

Despite the intensive research into microfluidics, an unexpectedly small number of POC diagnostic devices have been introduced commercially. We presume that the limited success of these POC devices is due to a reliance on the cost trade off of developing integrated disposable components versus historically more limited but lower cost non-microfluidic devices, and intellectual property limitations. In many devices, the lack of on-chip sample preparation further limits their usability as true POC devices. We only found two commercial systems that were capable of extracting and processing nucleic acids directly from biological samples. Micronics Inc. is advancing the PanNAT® System [252], a molecular diagnostic system capable of sample to result processing for multiplexed detection of bacteria, viruses and pathogens using real-time PCR amplification. The system uses a disposable microfluidic cartridge preloaded with all the necessary reagents and is able to provide results in approximately 60 min. Q-POC^TM^ [253] is a handheld molecular diagnostic device from QuantuMDx which can process genomic material from blood, tissue (fresh and FFPE), thinned sputum, and swabs. The system utilizes mechanical lysis, filters for extraction, continuous flow PCR amplification, nanowires sensors for detection uses cartridges containing pre-loaded dried reagents and probes for tuberculosis, malaria, HPV, chlamydia, and gonorrhea. Sample to result time is within 20 min. 

Several other systems require the preparation of nucleic acid samples prior to processing on the device. SRI International developed the Sentinel Nucleic Acid Analysis System [254] for low-cost diagnostic detection of bacteria, viruses, and mRNA. A single chip is capable of differentiating between 57 adenoviridae viruses from a single PCR amplicon. Other available systems appear to be targeted at routine genomic analysisas a research use only device. BioMark (Fluidigm) [255] and OpenArray (Life Technologies) [256] are digital microfluidic devices that partition samples for high-throughput gene expression. The SmartChip system (Wafergen) [257] comprises a nano dispenser module, thermal cycler, and data collection module to support PCR for high-density gene expression profiling. The RainDrop Instrument (RainDance Technologies) [258] is a dPCR platform capable of processing eight samples, where each of the samples is partitioned into 10 million reactions. The QX200 Droplet Digital PCR (ddPCR™) [259] by Bio-Rad System generates 20,000 water-emulsion nano-droplets with droplet fluorescence readout. 

## 9. Summary and Conclusions

In this paper, we have reviewed prominent microfluidic methods for cell lysis, nucleic acid extraction, amplification, and detection. Microfluidic diagnostic devices are not capable of mass-scale POC diagnosis of infectious pathogens. To achieve practical application at the POC, automated end-to-end genomic identification of a broad range of pathogens on a single device is needed.

The challenges lie mainly in the integration of all processing steps, taking special care to ensure compatibility and non-interference between processes and to guarantee specificity over a broad range of pathogens and a minimum detection limit. Lysis using chemicals requires the removal of chemicals; otherwise, they will inhibit the subsequent extraction and amplification process. Mechanical lysis requires complex fabrication steps and the geometry of structures is dependent on the size of the pathogen to be lysed. Thermal lysis requires the integration of heaters and control of thermal cycling. Regulating and cycling temperatures consumes energy, may degrade nucleic acids, and slows down the processing time. Electrical lysis can be battery-powered but may lead to Joule heating, which also degrades nucleic acids. 

The purity and efficiency of nucleic acid extraction is key to achieving high sensitivity and specificity; otherwise, the benefits of amplification are lost. However, only a handful of microfluidic systems are sufficiently robust and ready for direct amplification after lysis without extraction. Filtration-based extraction can be confounded by stray autofluorescence. Solid phase extraction methods require the immobilization of microbeads into a monolithic porous structure inside the channel. Magnetic-bead-based extraction requires binding and washing steps that increase the complexity, cost, and points of failure of the device. Isotachophoresis is dependent on the proper selection of both the electric field and electrolytes. Recent progress has shown that electrolytes can be made compatible with amplification techniques but are restricted in conductivity.

PCR is a widely used amplification technique. The stationary chamber-based PCR lacks time flexibility and requires precise control circuitry to regulate the different temperatures. Continuous flow PCR requires fine control of fluidic flow timing and regulation of reagent flow. The cyclic time and temperature also depend on the length of the base pair, amplicon, and primers. Isothermal amplification is an emerging alternative to PCR that is highly sensitive and more robust to the inhibitory chemical environment, but prone to mispriming and false positives from primer–dimer interactions.

Colorimetric detection that is visible to the naked eye is attractive but may have sufficient variation between samples to confound quantification or standardization of processing time. Fluorescence detection requires specialized and sensitive optical detection systems, an optically transparent device substrate, and special indicator molecules that will raise the device costs. Smartphone-based detection is emerging as a viable method for optical detection but still requires expensive optical filters. Electrochemical detection is influenced by temperature, pH, and ionic concentrations, thus limiting its sensitivity. 

Paper microfluidics is attractive because devices can be passively actuated and the material is cheap, disposable, and foldable. However, devices suffer from poor detection sensitivity and loss or contamination of samples. The fabrication of complex microfluidic structures and complex fluidic manipulations also remains a topic of research. Digital microfluidics has potential for complex, multi-target, multi-step analysis with precise actuation. However, devices are not reusable and the threat of sample contamination through insufficient cleaning and sample loss by evaporation is a major limitation. Centrifugal microfluidics utilizes the rotational mechanism of CD players, which is a common device and also easy to perform manually. However, designs are complex and it is unclear whether a solution integrating cell lysis and nucleic acid extraction that could perform robustly in POC settings will emerge. 

## 10. Future Outlook

We foresee that the advantages of using microfluidic molecular techniques will provide a great payback in the realization of low-cost multiplexed, highly sensitive, and specific diagnosis from a single sample with automated processing capabilities, enabling low power consumption. Devices should integrate the sample preparation steps suitable for simultaneous extraction and amplification of DNA and RNA from complex sources (e.g., blood, saliva, and urine). The primers designed for amplification should be universal and specific to cover a wide range of genetic variations. In addition, the reagents used in microfluidics device should be stable at room temperature with a longer shelf life. Moreover, the devices should be biodegradable along with infectious pathogens so as to avoid the risk of contamination and spreading of infectious diseases.
